# Laboratory measurements of viscosity, density, and bulk contact angle on marble and soda lime glass for three naphthenic acid + *n*-decane solutions

**DOI:** 10.1016/j.dib.2019.103988

**Published:** 2019-05-10

**Authors:** Xanat Zacarias-Hernandez, Magali Christensen, Yukie Tanino, Olalekan O. Ajayi

**Affiliations:** School of Engineering, University of Aberdeen, UK

## Abstract

This paper presents static oil/brine contact angles measured using the sessile drop method on soda lime glass and polished marble. Pure *n*-decane and three 66 mM naphthenic acid solutions in *n*-decane were considered as model oils. Selected naphthenic acids were: cyclohexanecarboxylic acid (CHCA), cyclohexanebutyric acid (CHBA), and cyclohexanepentanoic acid (CHPA); all oils were dyed with Oil Red O (ORO) at a concentration of 0.9 mM. Also presented are complementary density and viscosity measurements by rotational viscometry at selected temperatures ranging from *T* = 16.00–28.00 °C. For the application of the data to interpret microfluidic experiments, see Tanino et al. [1] and Christensen et al. [2].

**Table:** Specifications Table

Subject area	*surface chemistry*
More specific subject area	*wettability, interfacial properties*
Type of data	*raw images, Excel tables, Excel spreadsheet*
How data were acquired	*contact angles: Nikon SMZ745T optical microscope; Pixelink PL-B742F camera;* *viscosity, density: Anton Paar SVM*^*TM*^*3000*
Data format	*raw, analyzed*
Experimental factors	*Static oil/brine contact angle measurements on soda lime glass and polished marble using the sessile drop method.*
Experimental features	*The substrates were pre-equilibrated in brine, then treated with naphthenic acid + n-decane solutions. Density and dynamic viscosity of the test fluids were measured at T = 16 to 28°C.*
Data source location	*With corresponding author at University of Aberdeen,* *Aberdeen, UK.*
Data accessibility	*Data is with this article*
Related research article	M. Christensen, X. Zacarias-Hernandez & Y. Tanino (2018) Secondary waterflood under mixed-wet conditions: crossover from stable displacement to capillary fingering in a microfluidic packed bed. *Advances in Water Resources*, under revision.

**Table:** 

Value of the Data • The contact angle data can be used to evaluate the wettability/hydrophilicity of organic acid-treated soda lime glass and marble. • The data can be used to evaluate the suitability of naphthenic acid solutions as analogues of crude oil or NAPL in research in the area of multiphase porous media flow. • The raw images may be used as test images to develop and validate algorithms for extracting contact angles. • The data can be compared against *in situ* oil/brine contact angles in porous media (e.g., carbonate rock) measured using, e.g., X-ray micro-computed tomography.

## 1 Data

The dataset comprises (a) static oil/brine contact angles measured using the sessile drop method and (b) thermophysical properties of the test fluids. In total, 63 sessile drops on two substrates (soda lime and marble) submerged in one of four oils (n-decane + Oil Red O (ORO) or one of three 66 mM naphthenic acid solutions in n-decane + ORO) were analyzed; raw images (one per drop) can be found in the Supplementary Materials. The extracted contact angles are reported in Excel file S2 as a function of aging time in one of two spreadsheets: one for marble (34 contact angles; spreadsheet ‘marble’) and one for soda lime (84 contact angles; spreadsheet ‘soda lime’). Excel file S1 lists measured density and dynamic viscosity at selected temperatures between T = 16 and 28 °C; Table 1 presents empirical models that describe the temperature dependence of the density and viscosity.

**Table 1:** Lines of best fit in the least squares sense to density and viscosity measurements (Excel file S1). The best-fit functions were previously used to evaluate the density and viscosity of brine and *n*-decane + ORO at *T* = 21 °C [1], [2], [3], [4]. The brine is a solution of 5.0 wt% NaCl, 1.0 wt% KCl in deionized water saturated with carbonate. The best-fit function for the viscosity of the test brine was previously reported in Ref. [5].

test fluid	density [kg/m^3^]	viscosity [*μ*Pa s]
*n*-decane + ORO	(745.60 ± 0.003) - (749.9 ± 0.1) x 10^−3^*T*	(1131 ± 6) - (13.1 ± 0.3) x 10^−3^*T*
66 mM CHCA + ORO	(748.74 ± 0.1) - (757 ± 4) x 10^−3^*T*	(1152 ± 10) - (13.4 ± 0.5) x 10^−3^*T*
66 mM CHBA + ORO	(749.2 ± 0.1) - (772 ± 7) x 10^−3^*T*	(1180 ± 11) - (14.0 ± 0.5) x 10^−3^*T*
66 mM CHPA + ORO	(748.87 ± 0.08) - (760 ± 4) x 10^−3^*T*	(1232 ± 17) - (15.8 ± 0.8) x 10^−3^*T*
brine [5]	(1047.3 ± 0.2) - (342 ± 10) x 10^−3^*T*	(1589 ± 13) - (22.8 ± 0.6) x 10^−3^*T*

## 2 Experimental design, materials, and methods

### 2.1 Test fluids

The aqueous phase was a 5.0 wt% NaCl and 1.0 wt% KCl solution in deionized water (Milli-Q Direct 8, Millipore) equilibrated with crushed limestone on a magnetic stirrer for a minimum of 48 h (e.g., Ref. [6]).

Four oils were considered: *n*-decane (Sigma Aldrich ReagentPlus ≥99%) and 66 mM solutions of cyclohexanecarboxylic acid (CHCA; Sigma-Aldrich 98%), cyclohexanebutyric acid (CHBA; Sigma-Aldrich 99%), and cyclohexanepentanoic acid (CHPA; Sigma-Aldrich 98%) in *n*-decane. All oils were dyed with Oil Red O (ORO; powder, certified by Biological Stain Commission, Sigma-Aldrich), a lysochrome (oil-soluble) diazo dye. The solutions were made as follows, at ambient temperature:

1. Powdered ORO was added to *n*-decane at a concentration of 0.9 mM and mixed well in a flask with a lid.
2. The resulting translucent red solution was filtered through a sheet of grade 42 filter paper (2.5 μm particle retention, Whatman) to remove any undissolved powder.
3. Solutions of organic acid listed above were prepared by adding the corresponding acid to aliquots of the filtered solution to yield a concentration of 66 mM.

### 2.2 Density and dynamic viscosity measurements

Dynamic viscosities and densities of each test fluid were measured by rotational viscometry at selected temperatures between *T* = 16.00 and 28.00 °C (Anton Paar SVM™ 3000). Over this range of *T*, the densities and the corresponding dynamic viscosities of each test fluid decrease linearly with increasing *T*, and a linear function of the form:

$$\begin{array}{c} \rho \\ \mu \end{array}\}=A+B\;T\;[{}^{\circ}C]$$

was fitted to data in the least-squares sense. The uncertainty in the best-fit coefficients A and B were calculated as ([7], Eqs. 8.12, 8.15–8.17):

$$\delta_{A}=\sqrt{\frac{1}{N-2}\overset{N}{\underset{m=1}{\sum }}{\left[y_{m}-\left(A+B\;T_{m}\right)\right]}^{2}}\sqrt{\frac{\sum_{m=1}^{N}{T_{m}}^{2}}{N\sum_{m=1}^{N}{T_{m}}^{2}-{\left(\sum_{m=1}^{N}T_{m}\right)}^{2}}}$$

and

$$\delta_{B}=\sqrt{\frac{1}{N-2}\overset{N}{\underset{m=1}{\sum }}{\left[y_{m}-\left(A+B\;T_{m}\right)\right]}^{2}}\;\sqrt{\frac{N}{N\sum_{m=1}^{N}{T_{m}}^{2}-{\left(\sum_{m=1}^{N}T_{m}\right)}^{2}}}$$

respectively, where (*T*_m_, *y*_m_) denote a single measurement of viscosity (*y* = *μ*) or density (y=ρ), *m* = 1, 2, …, *N*, and *N* is the total number of data points; in the present experiments, *N* = 4 for each test oil. Note that $\delta_{A},\;\delta_{B}$ reflect the deviation of data from a linear dependence on *T* [7]. The functions for the lines of best-fit are presented in Table 1. Note that the corresponding data for the same oils, but without ORO, are reported in Ref. [5].

### 2.3 Contact angle measurements

#### 2.3.1 Substrate preparation

Soda lime substrates were prepared by cutting 0.96–1.06 mm thick glass slides (Corning^®^ microscope slides, plain) to 10 mm × 15 mm using a wheel glass cutter. Marble substrates were prepared by cutting a block of white Carrara marble, Italy to roughly 15 mm × 15 mm x 5 mm pieces, then polishing them with a suspension of *MicroPolish II alumina suspension*, 0.3 μm (Buehler) in distilled water and a *TexMet C* polishing cloth (Buehler). After cutting and polishing, the substrates were flushed with toluene, then 2-propanol, and finally deionized water.

The substrate was first submerged in brine for 15–28 h at room temperature (*T* = 19.9–21.0 °C), then submerged in the test oil in a glass cuvette (Hellma Analytics 704-OG, internal dimensions 20 × 20 × 20 mm).

#### 2.2.2 Sessile drop method

After a pre-selected ageing time ranging from *t*_a_ = 1–96 h, a drop of brine (approx. 6–10 μL) was manually dispensed onto the substrate and imaged from the side using a 1280 × 1024 colour camera (Pixelink PL-B742F) coupled to a trinocular microscope (Nikon SMZ745T). An objective magnification of 3xwas used to image soda lime/oil/brine systems and either 1x, 1.25x, 1.5x, 3x, or 5x for marble/oil/brine. Combined with a 0.55x built-in C-mount magnification, these values yield image pixel sizes ranging from 2.44 to 12.18 μm/pix (Table 2).

**Table 2:** Microscope zoom and the corresponding image pixel size.

microscope zoom	image pixel size [*μ*m]
1x	12.18
1.25x	9.75
1.5x	8.12
3x	3.05
5x	2.44

#### 2.2.3 Camera alignment

**Fig. 1:**
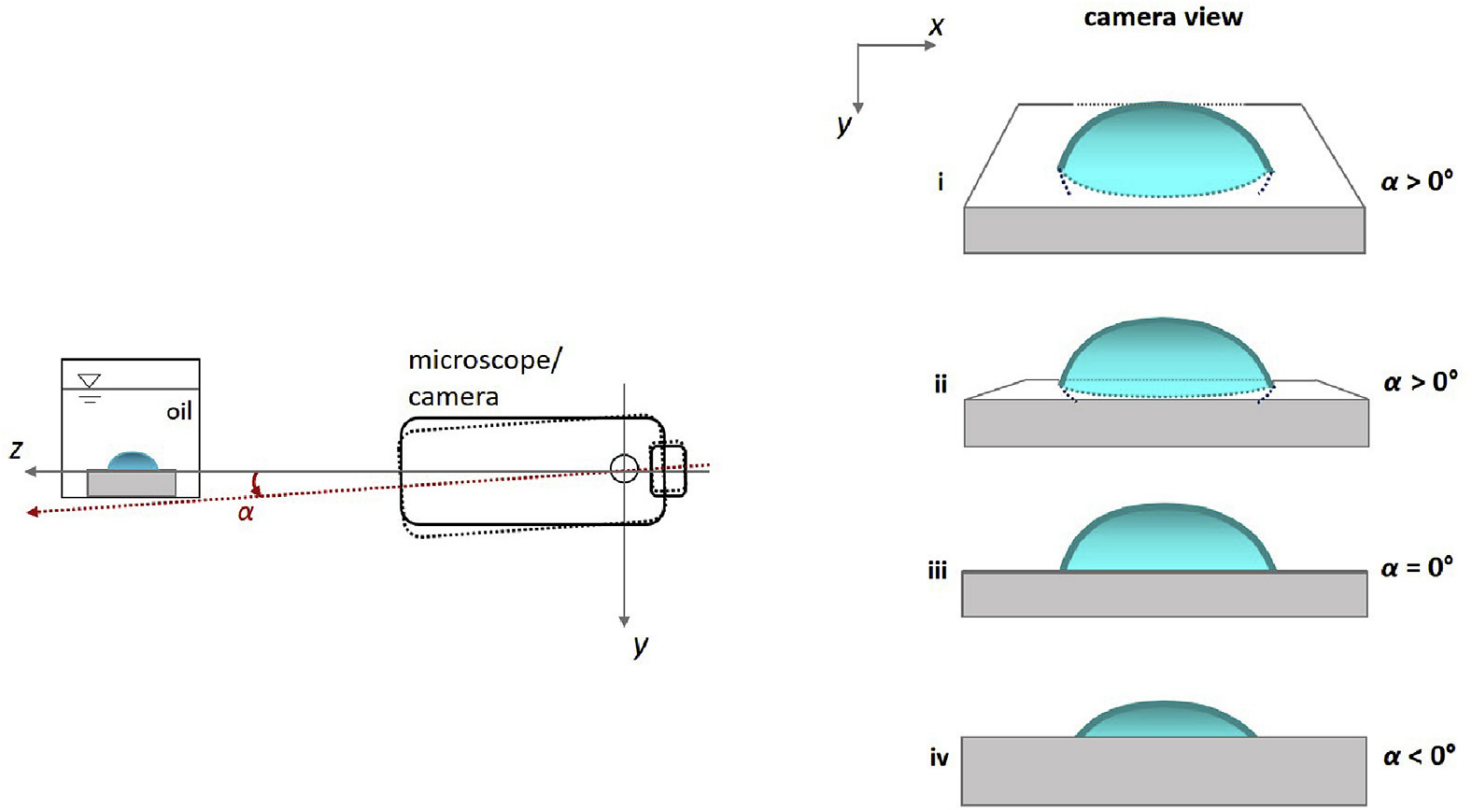
Fine adjustment of the camera alignment. After the coarse alignment of the camera and microscope with the upper plane of substrate (α=0), the camera/microscope is rotated slightly so it is looking down on the substrate (dotted lines; α>0). Then, gradually, the camera/microscope is rotated towards the horizontal until it is horizontal (solid lines). Right: the substrate as captured by the camera at different *α*. Not to scale.

The coupled microscope/camera (hereafter camera) were aligned to the triple contact points in three steps. First, the cuvette was placed on a laboratory jack so that the top of the substrate was horizontal and level with the camera and the camera was focused on the cross-section of the sessile drop. Next, the camera was inclined downwards slightly such that the top plane of the substrate was visible (Fig. 1, scenario i). Then, the camera was gradually rotated back towards the horizontal until the top plane of the substrate just disappeared from view (Fig. 1, scenario iii, e.g., m_carboxylic_93.48h.tif).

The above approach ensures that the camera never imaged the sessile drop from below the top plane of the substrate, which would preclude the (true) triple contact points from being imaged (Fig. 1, scenario iv). Where perfect alignment could not be achieved due to limitations on the precision with which the camera could be rotated, the camera was rotated conservatively and the reflection of the drop profile on the substrate could be discerned (scenario ii, e.g., sl_pentanoic_2.10h.tif).

#### 2.3.4 Experimental conditions

Between 2 and 4 drops were dispensed on different areas of the substrate. During the imaging, the drop was lit from the back using a desk lamp. A single drop was captured per image for all experiments on soda lime and many of the experiments on marble. Images in which more than one drop was captured have been cropped for clarity (Supplementary Materials).

The experiment was performed entirely in a temperature-controlled laboratory (*T* =20 to 21 °C). The temperature of the substrate was measured using an infrared thermometer (Digi-Sense Traceable^®^ IR Thermometer) at selected times during each stage of the experiment. During brine pre-equilibration, the temperature was measured twice: immediately after substrate immersion and immediately before transferring the substrate into oil. During ageing, the temperature was recorded when the substrate was first immersed in oil and again before the brine drop was dispensed; where *t*_a_ ≥ 70 h additional measurements were taken approximately every 24 h. After *t*_a_, the *in situ* temperature was recorded when the drop was dispensed and immediately after image acquisition.

#### 2.3.5 Determination of contact angle from the images

Two contact angles – one on either side of the drop – were extracted from each image by fitting, in the least-squares sense, a fourth-order polynomial function to the drop interface in polar coordinates following the protocol of Atefi et al. [8]; the drop interface was extracted using the Canny method for edge detection [9] as implemented in MATLAB^®^. If the edge detection algorithm did not identify the drop interface within 45 μm of a triple contact point, a contact angle was not calculated at that contact point. Oil/brine contact angles measured on marble and soda lime substrates are tabulated in Supplementary Materials.^1^

## Footnotes

1 Dynamic advancing contact angles on polished calcite for the same oil/brine pairs, but without ORO, are reported in Ref. [10].
