# Supplementary figures and images for: Laboratory measurements of viscosity, density, and bulk contact angle on marble and soda lime glass for three naphthenic acid + n-decane solutions
Source: Data Brief. 2019 May 10;24:103988. doi: 10.1016/j.dib.2019.103988 (PMC6536672; doi:10.1016/j.dib.2019.103988)

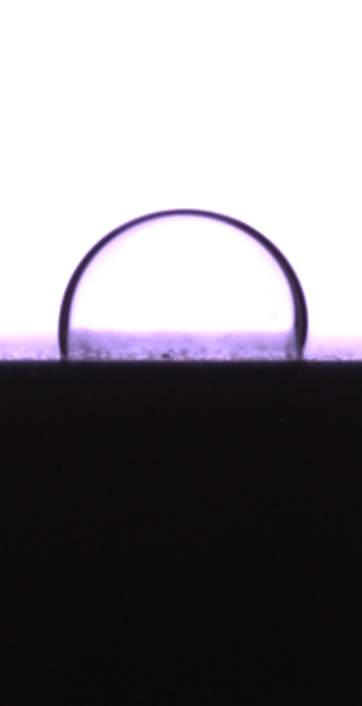

Supplement: Multimedia component 3 [file mmc3.zip › m_butyric_1.05h.tif]

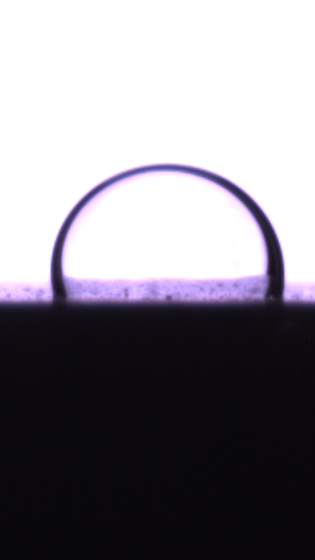

Supplement: Multimedia component 3 [file mmc3.zip › m_butyric_1.28h.tif]

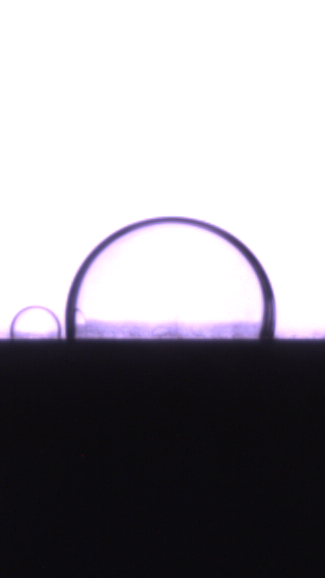

Supplement: Multimedia component 3 [file mmc3.zip › m_butyric_2.07h.tif]

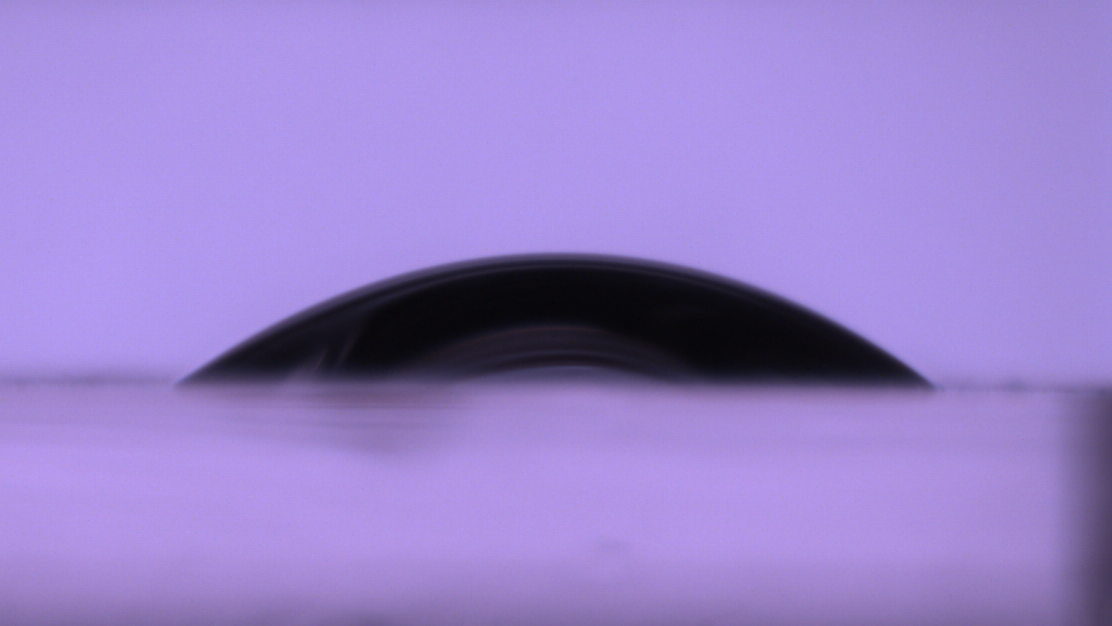

Supplement: Multimedia component 3 [file mmc3.zip › sl_butyric_1.45h.tif]

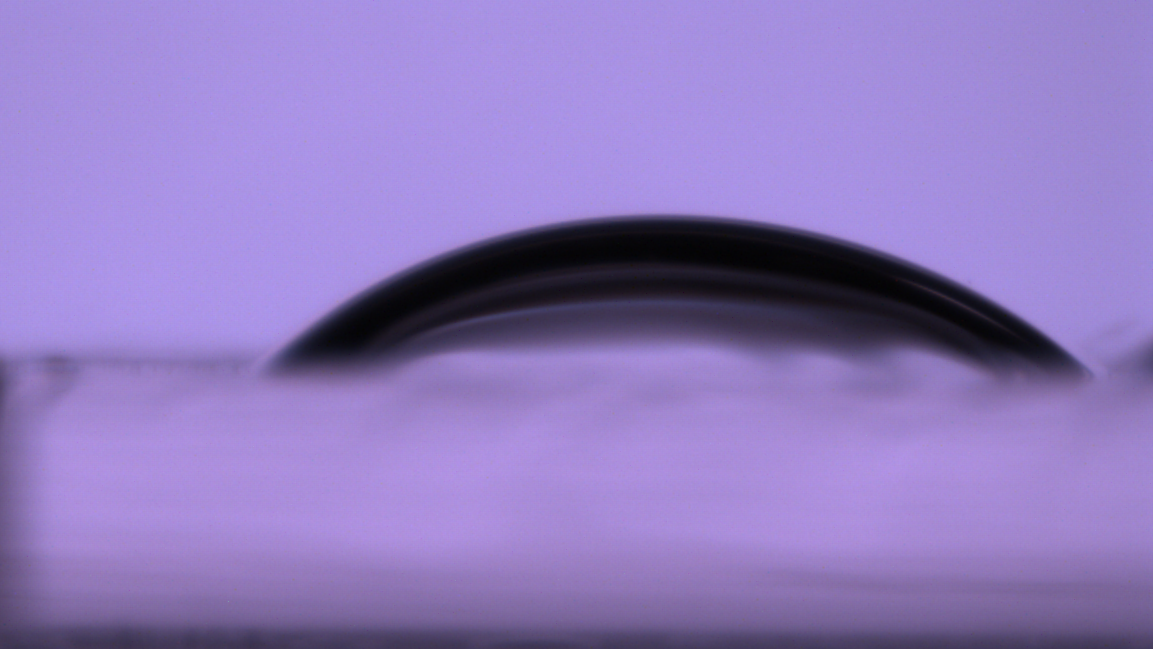

Supplement: Multimedia component 3 [file mmc3.zip › sl_butyric_1.55h.tif]

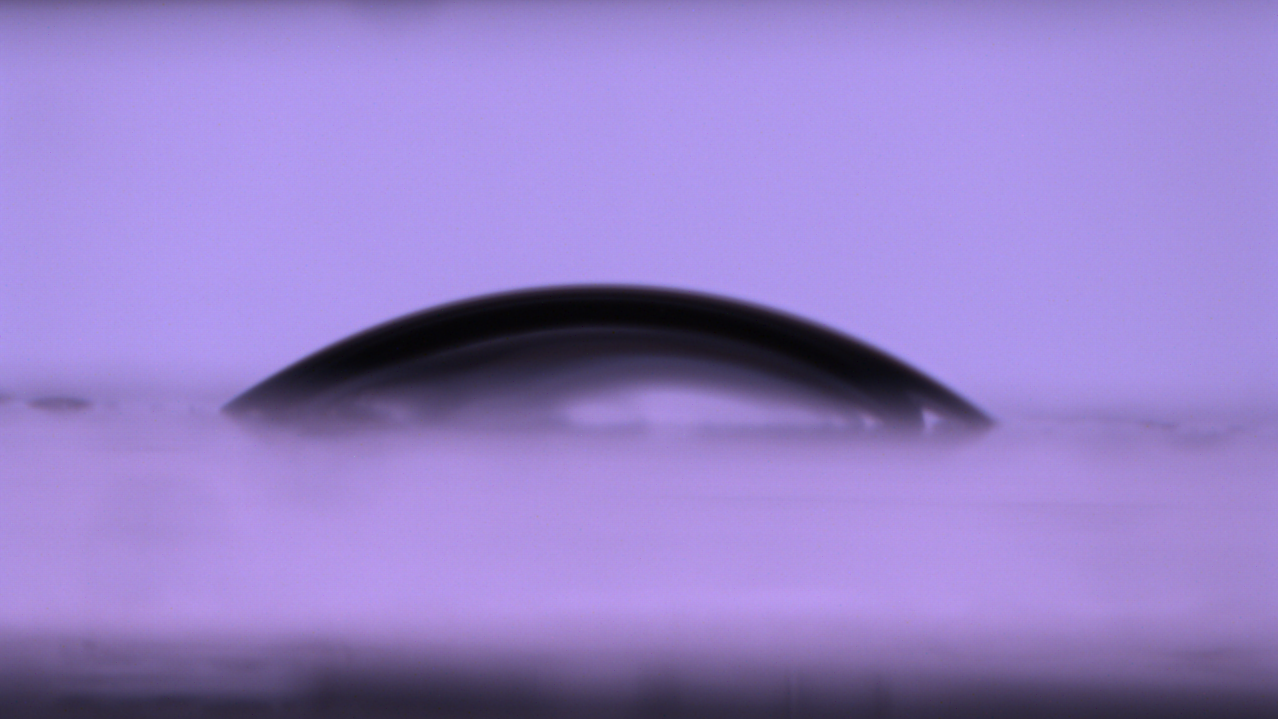

Supplement: Multimedia component 3 [file mmc3.zip › sl_butyric_1.72h.tif]

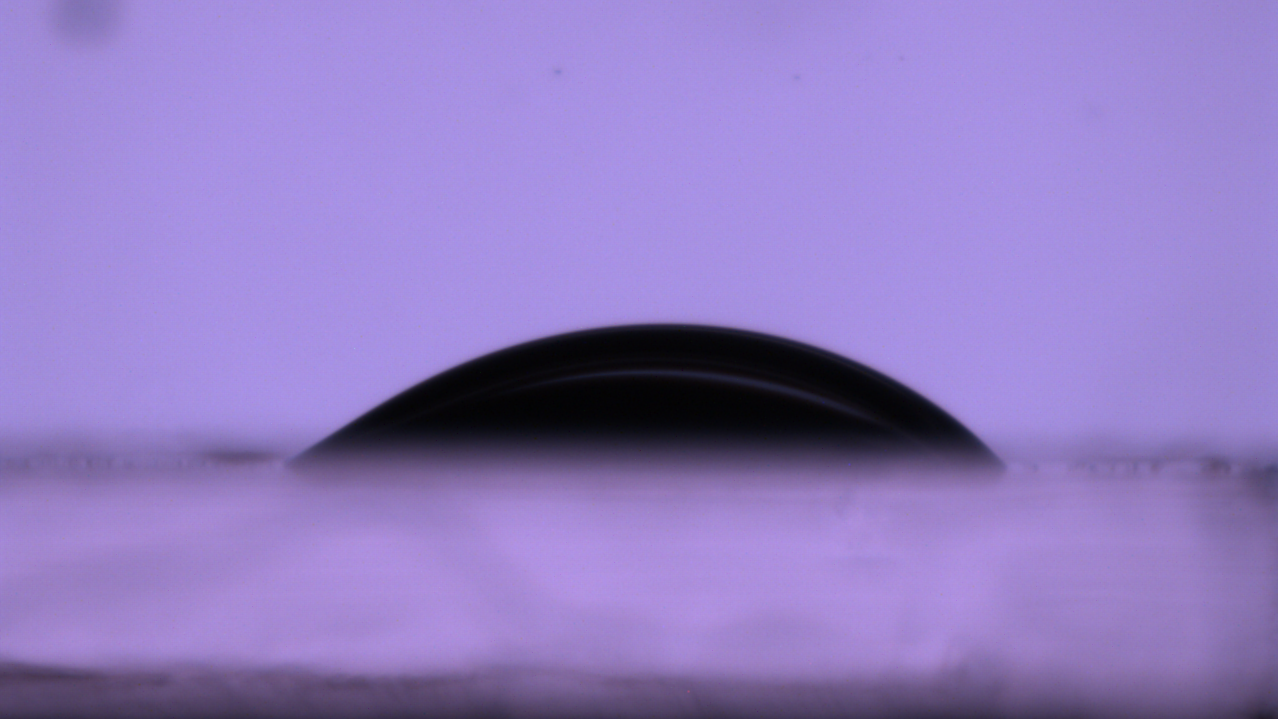

Supplement: Multimedia component 3 [file mmc3.zip › sl_butyric_1.90h.tif]

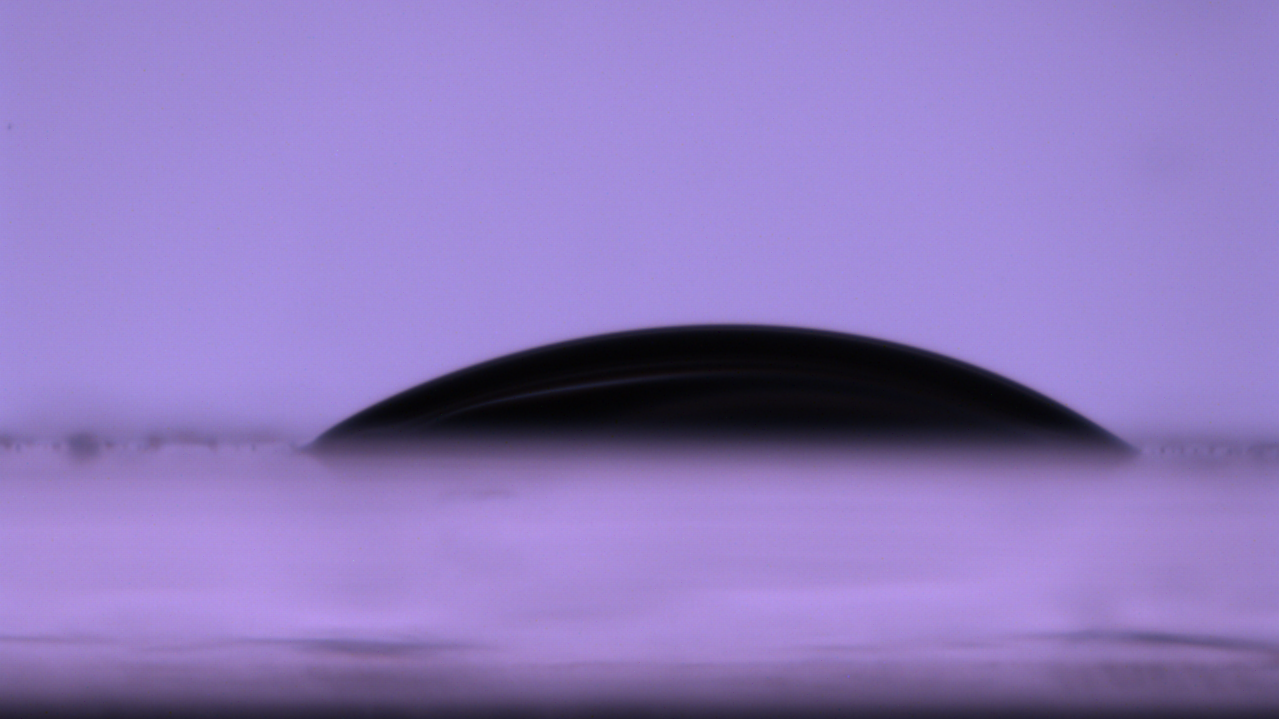

Supplement: Multimedia component 3 [file mmc3.zip › sl_butyric_1.97h.tif]

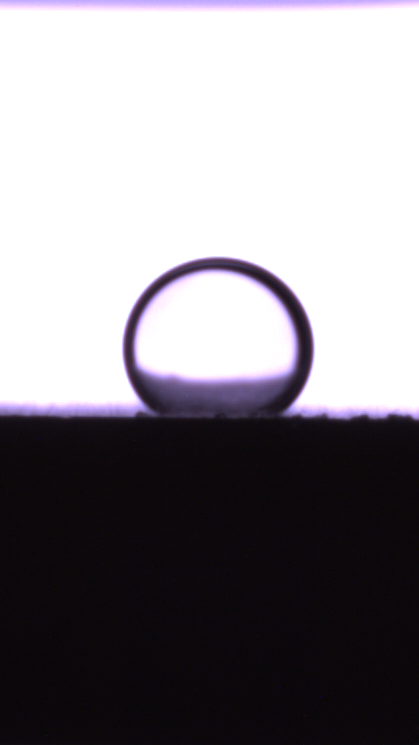

Supplement: Multimedia component 4 [file mmc4.zip › m_carboxylic_93.22h.tif]

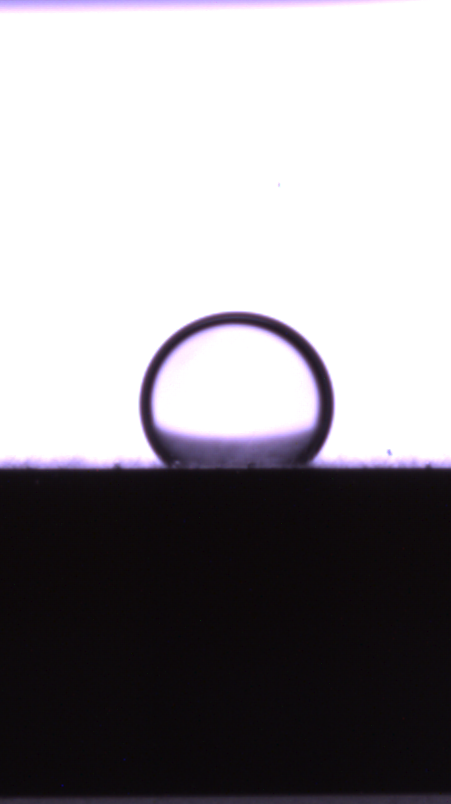

Supplement: Multimedia component 4 [file mmc4.zip › m_carboxylic_93.30h.tif]

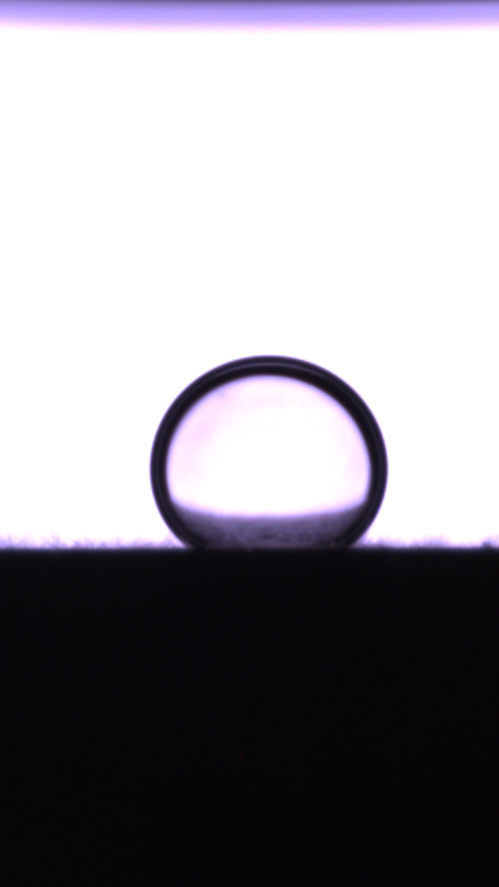

Supplement: Multimedia component 4 [file mmc4.zip › m_carboxylic_93.48h.tif]

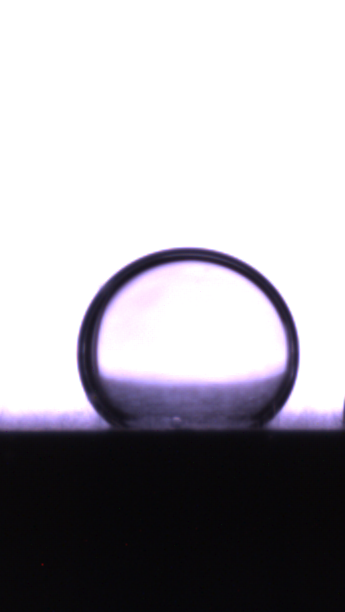

Supplement: Multimedia component 4 [file mmc4.zip › m_carboxylic_95.65h.tif]

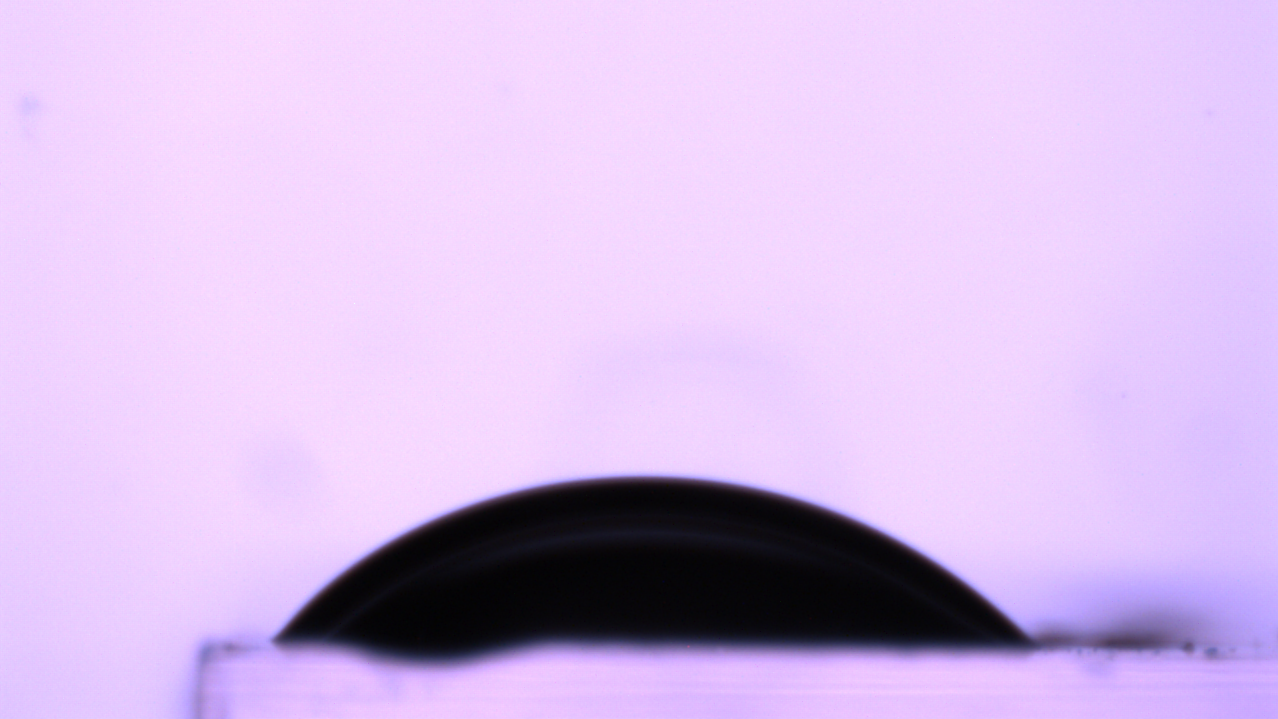

Supplement: Multimedia component 4 [file mmc4.zip › sl_carboxylic_75.38h.tif]

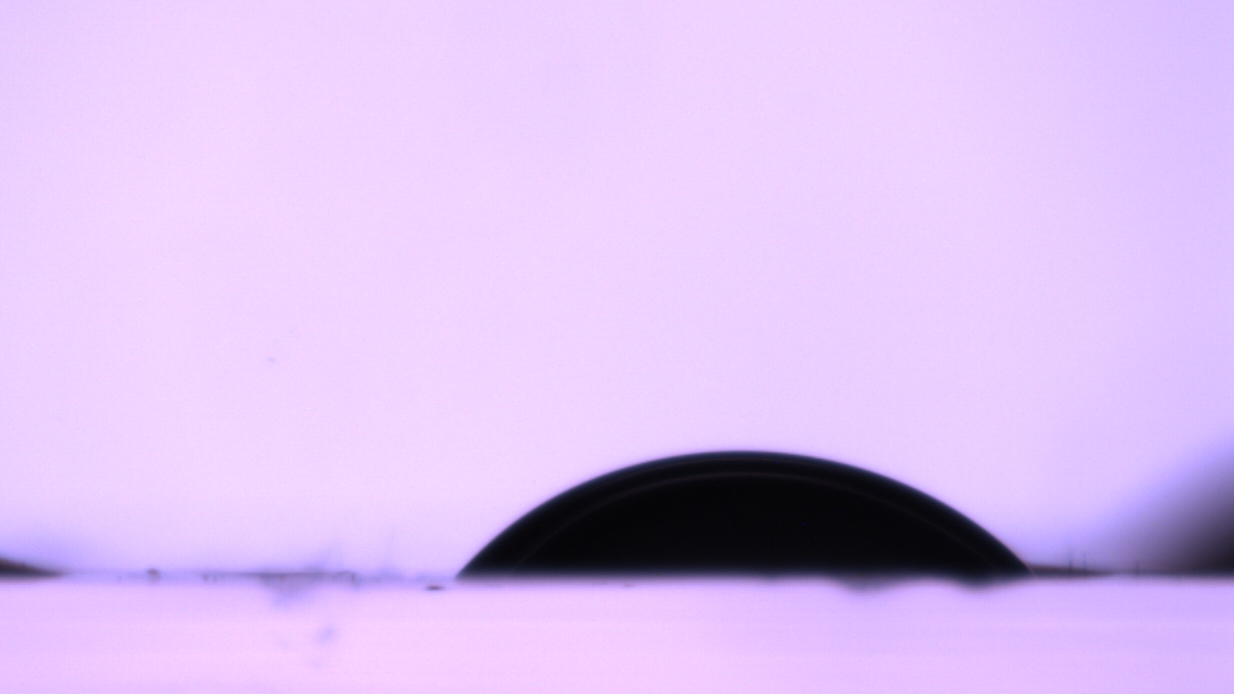

Supplement: Multimedia component 4 [file mmc4.zip › sl_carboxylic_75.48h.tif]

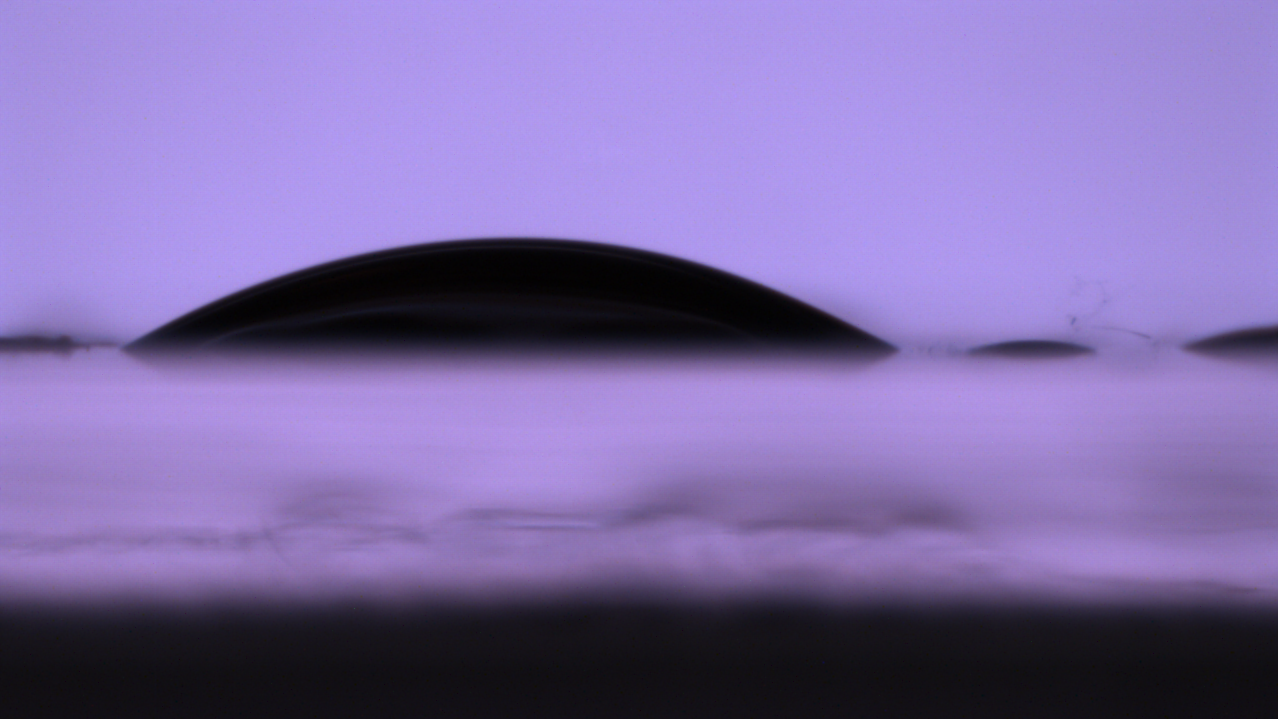

Supplement: Multimedia component 4 [file mmc4.zip › sl_carboxylic_75.65h.tif]

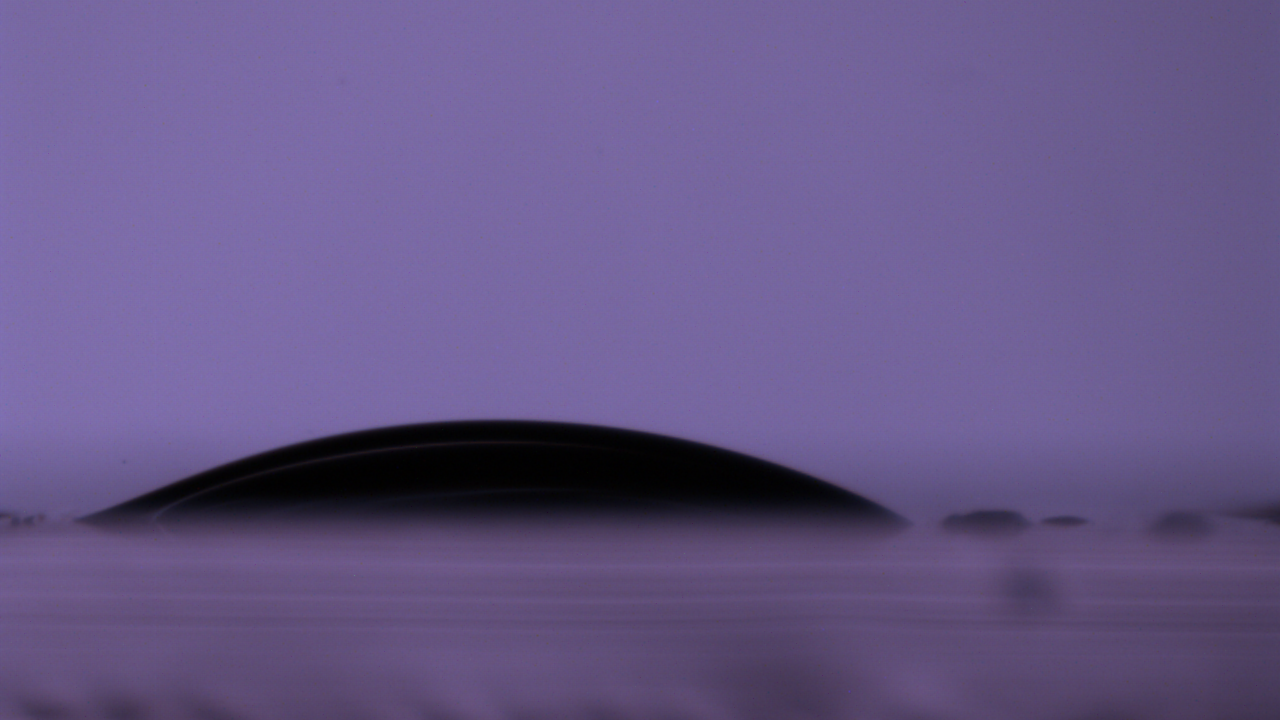

Supplement: Multimedia component 4 [file mmc4.zip › sl_carboxylic_75.73h.tif]

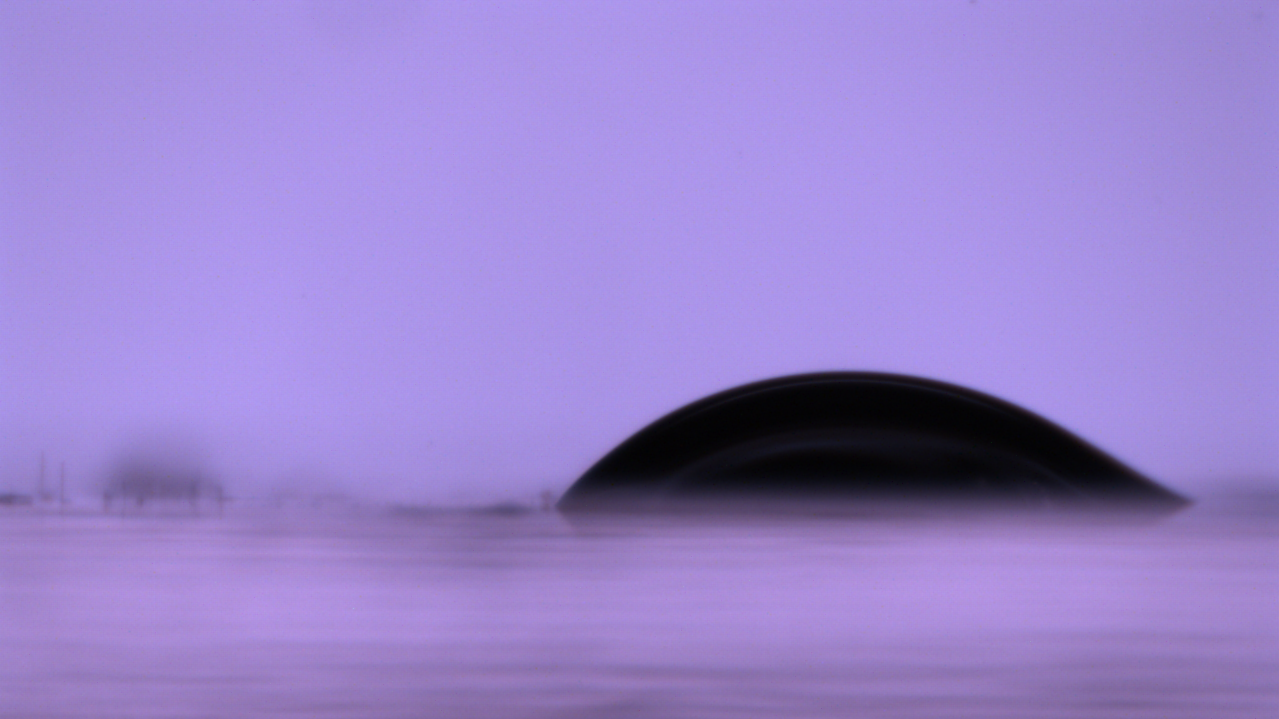

Supplement: Multimedia component 4 [file mmc4.zip › sl_carboxylic_75.85h.tif]

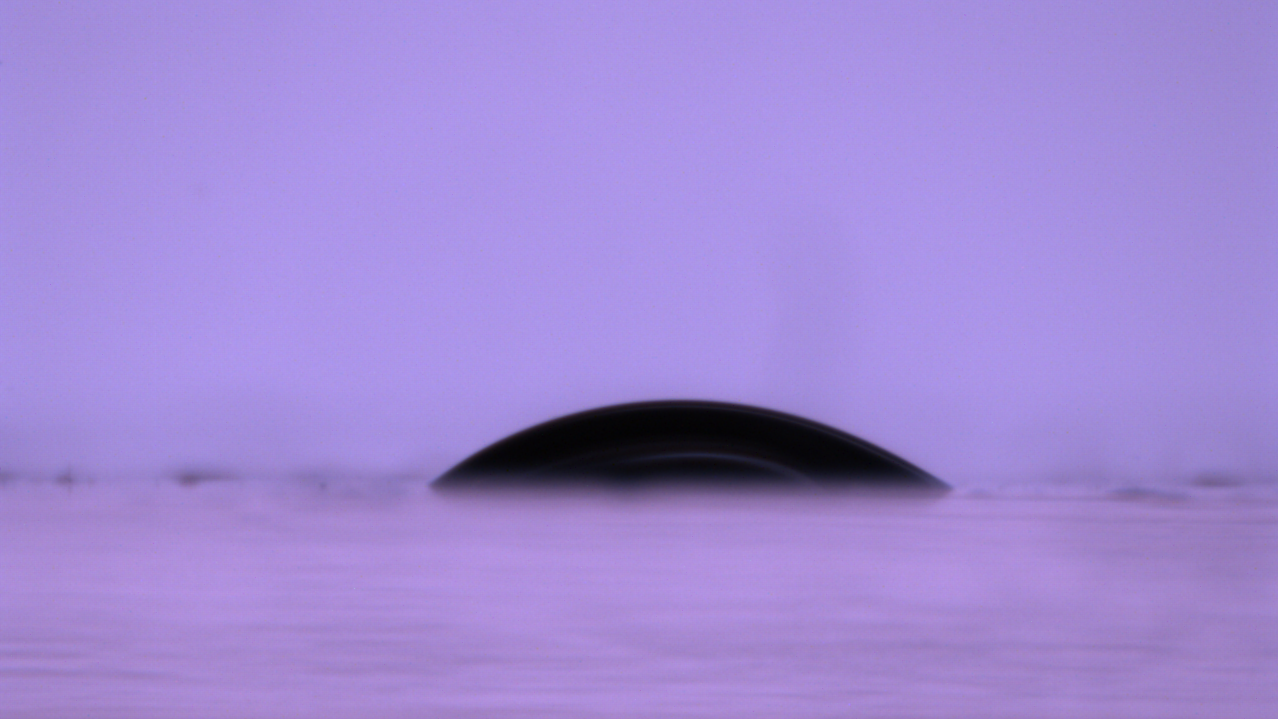

Supplement: Multimedia component 4 [file mmc4.zip › sl_carboxylic_75.88h.tif]

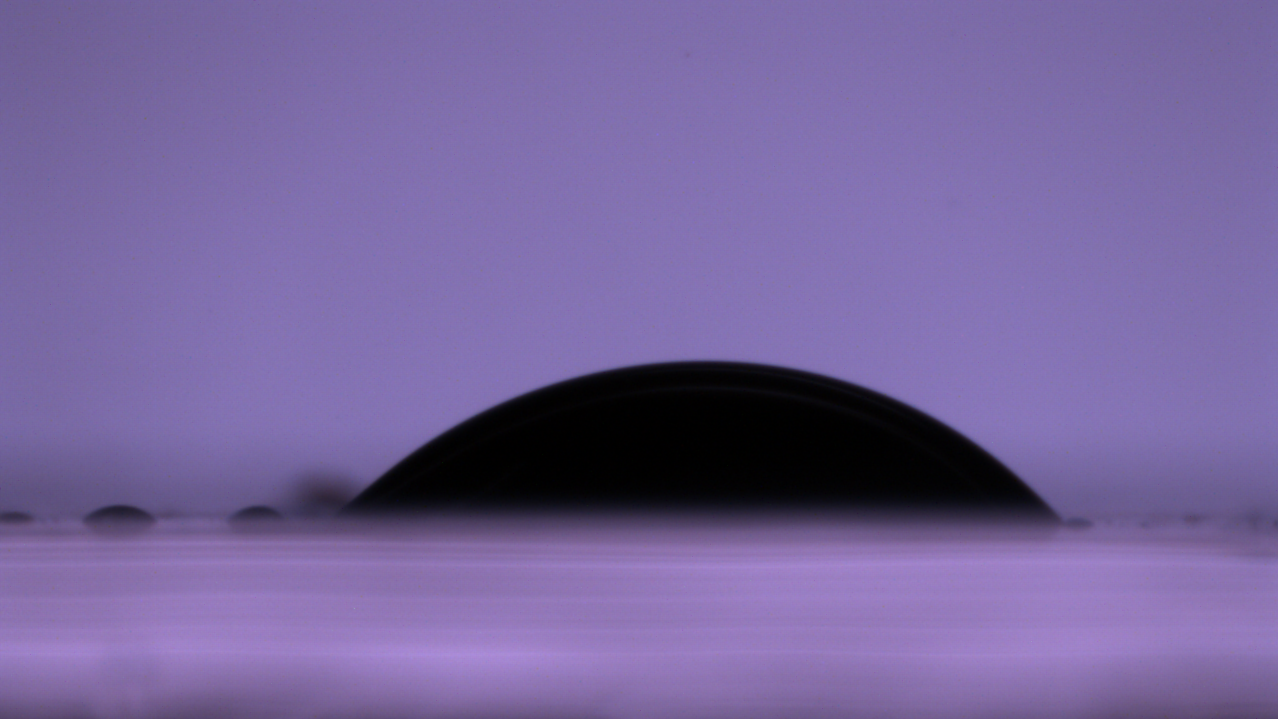

Supplement: Multimedia component 4 [file mmc4.zip › sl_carboxylic_75.92h.tif]

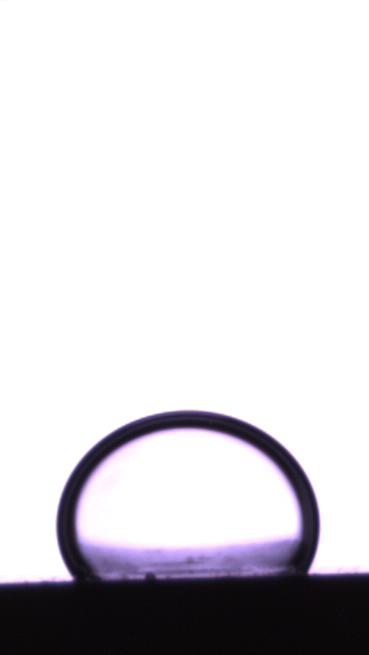

Supplement: Multimedia component 5 [file mmc5.zip › m_pentanoic_1.00h(2).tif]

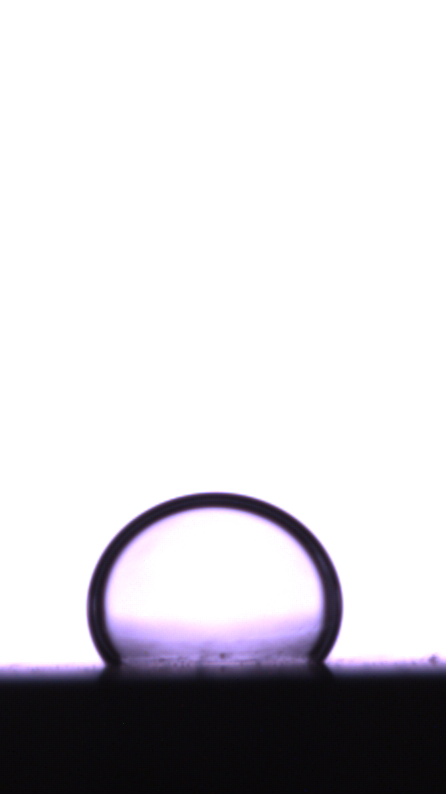

Supplement: Multimedia component 5 [file mmc5.zip › m_pentanoic_1.25h.tif]

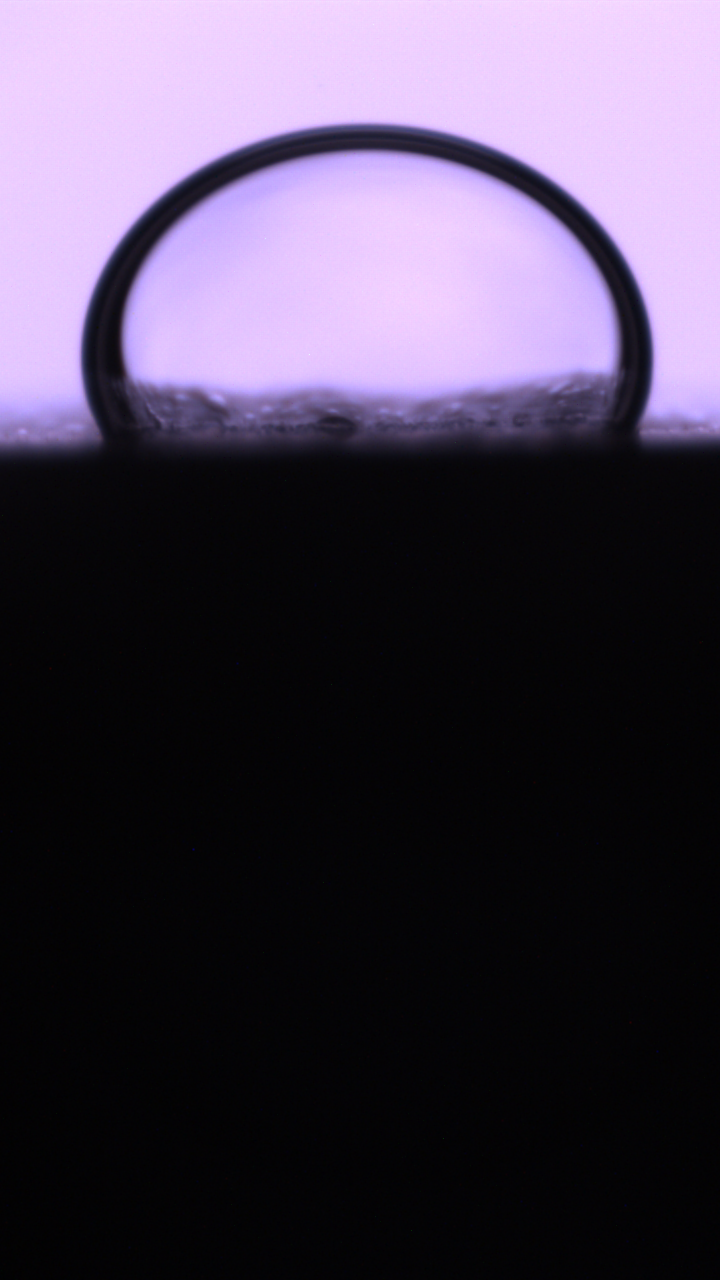

Supplement: Multimedia component 5 [file mmc5.zip › m_pentanoic_74.50h.tif]

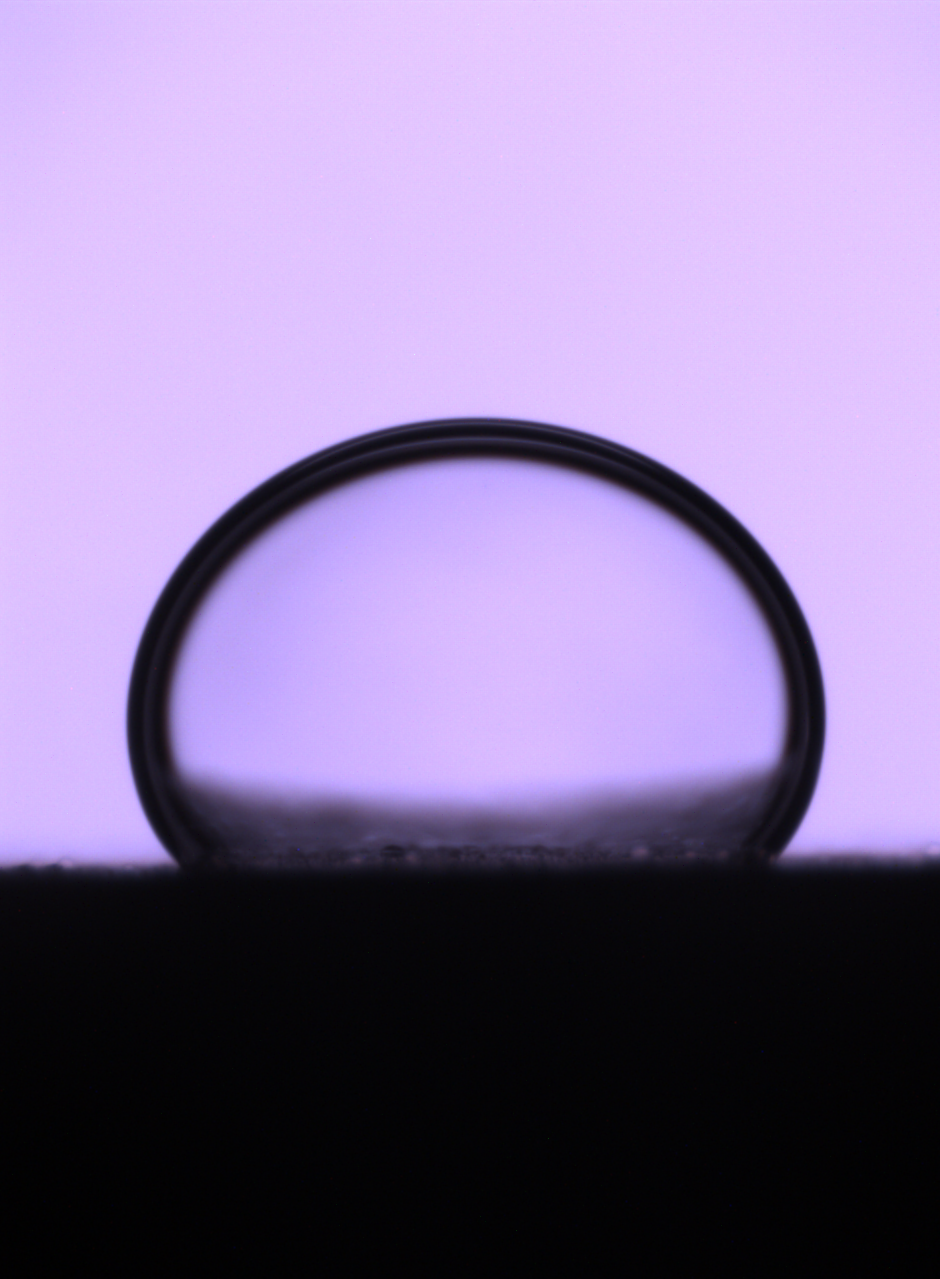

Supplement: Multimedia component 5 [file mmc5.zip › m_pentanoic_75.00h.tif]

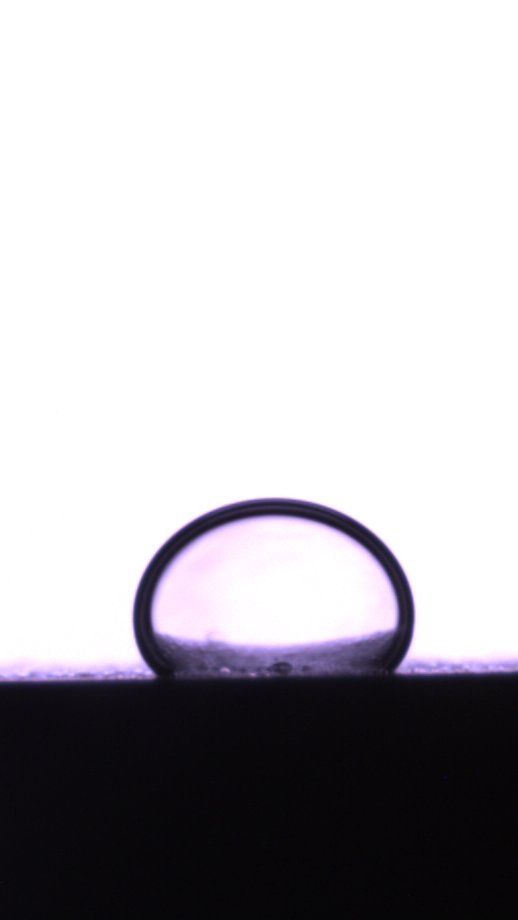

Supplement: Multimedia component 5 [file mmc5.zip › m_pentanoic_77.00h.tif]

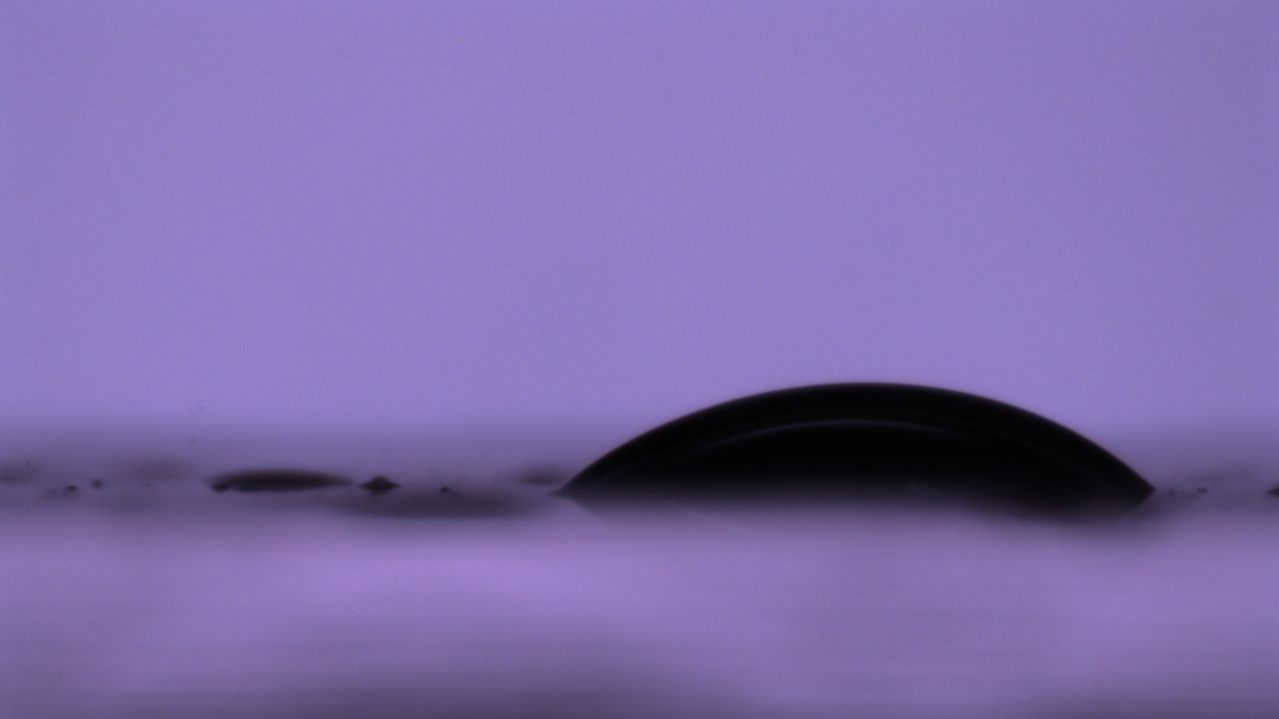

Supplement: Multimedia component 5 [file mmc5.zip › sl_pentanoic_1.63h.tif]

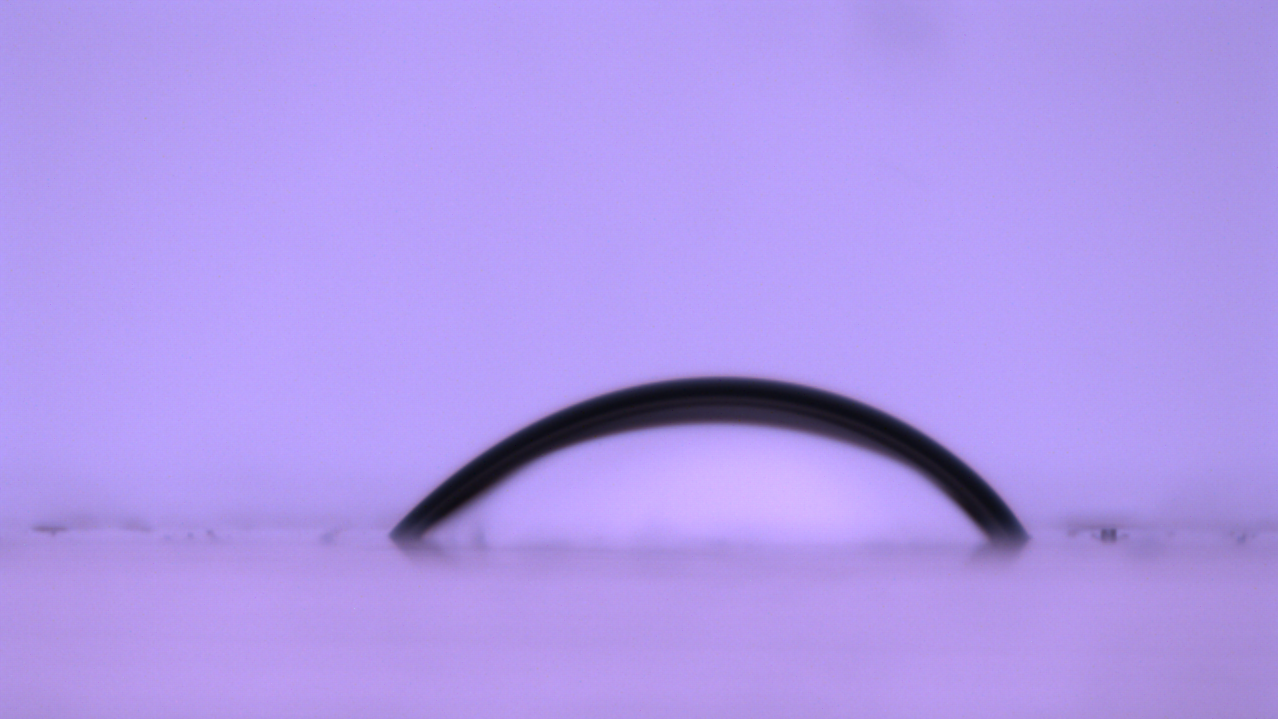

Supplement: Multimedia component 5 [file mmc5.zip › sl_pentanoic_1.70h.tif]

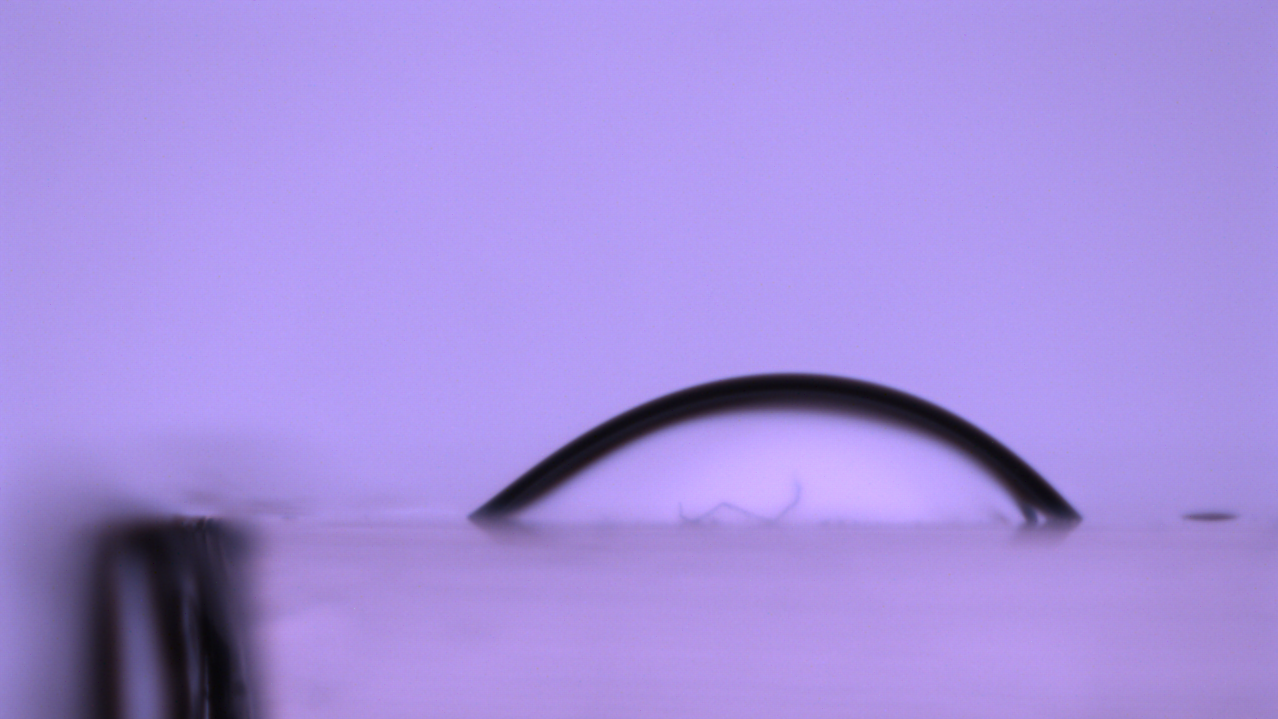

Supplement: Multimedia component 5 [file mmc5.zip › sl_pentanoic_1.75h.tif]

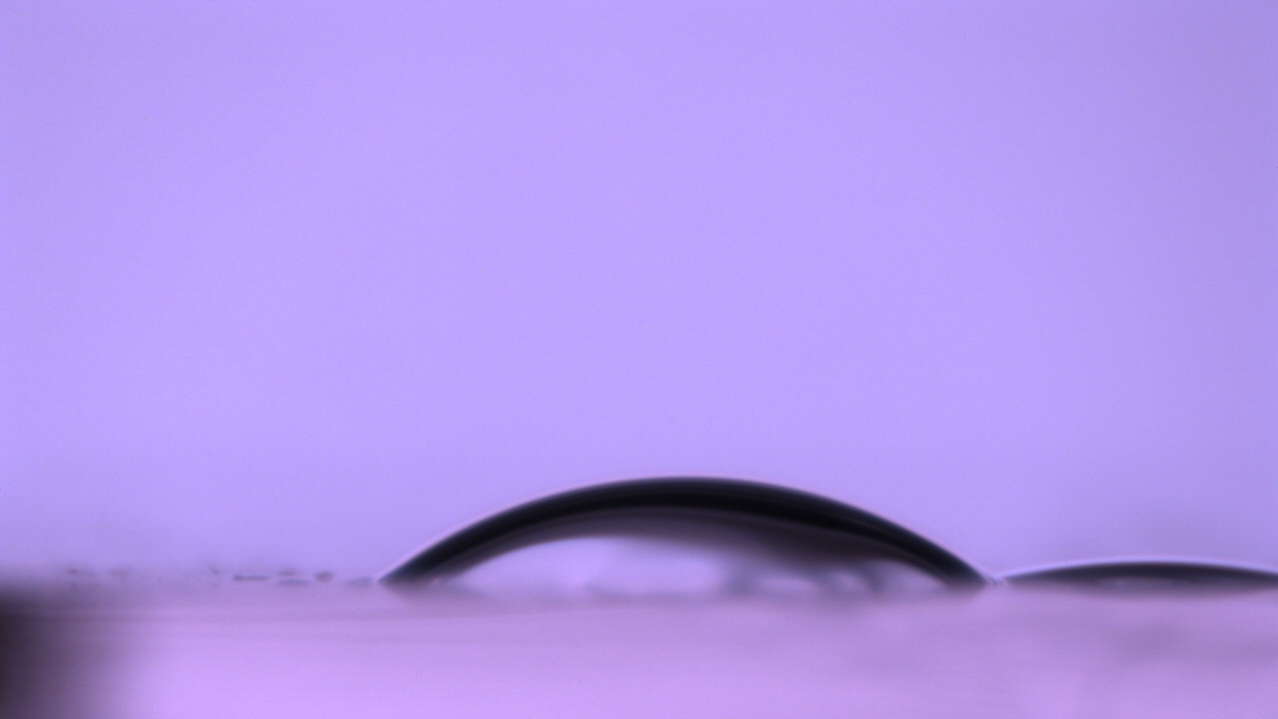

Supplement: Multimedia component 5 [file mmc5.zip › sl_pentanoic_1.80h.tif]

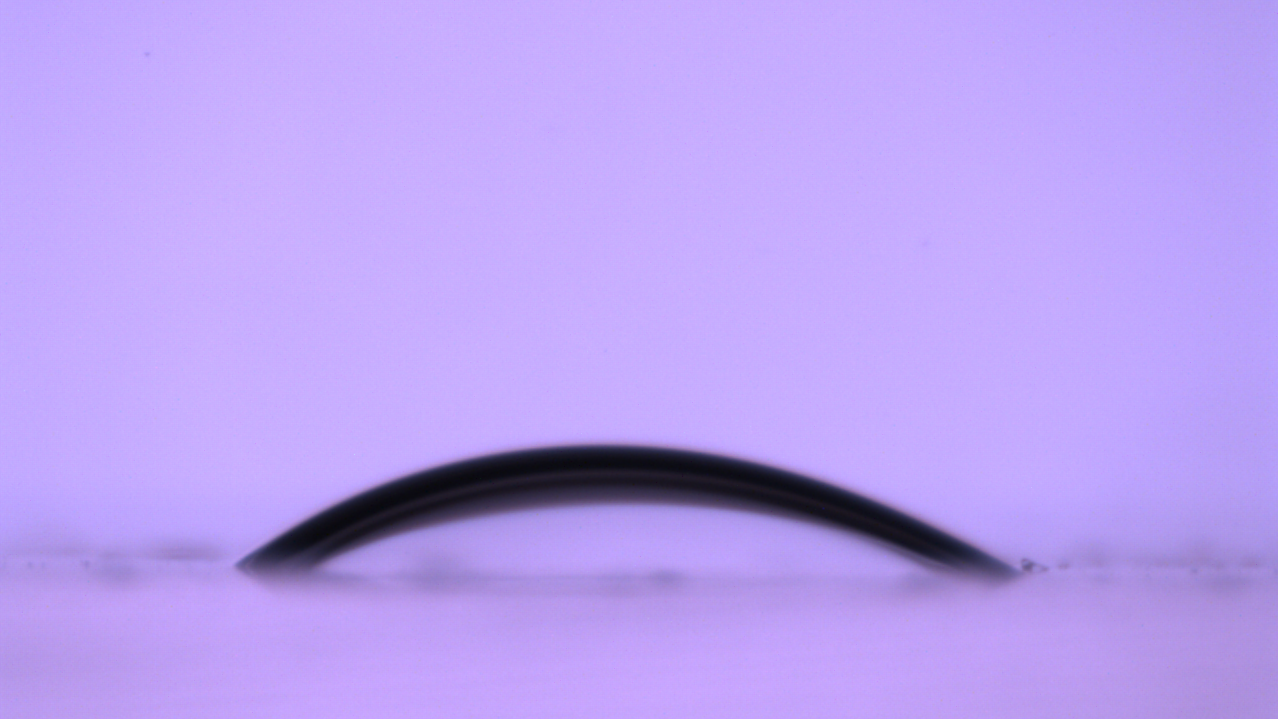

Supplement: Multimedia component 5 [file mmc5.zip › sl_pentanoic_1.98h.tif]

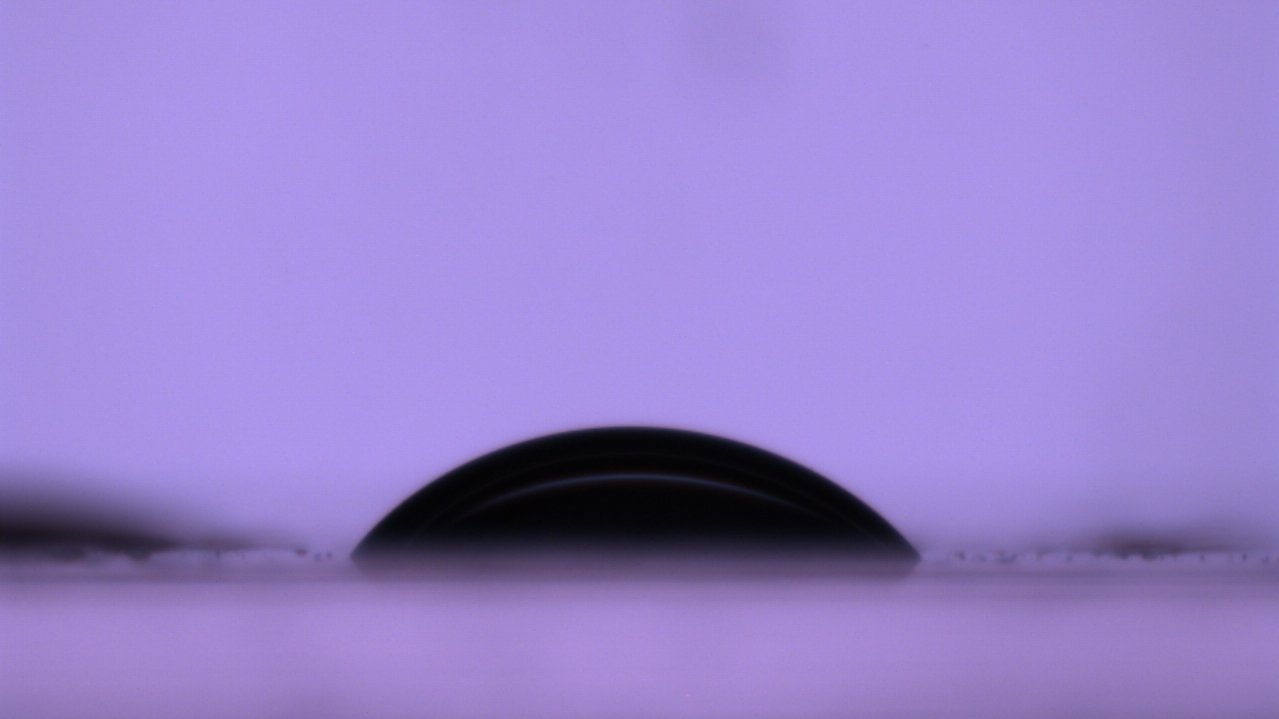

Supplement: Multimedia component 5 [file mmc5.zip › sl_pentanoic_2.10.tif]

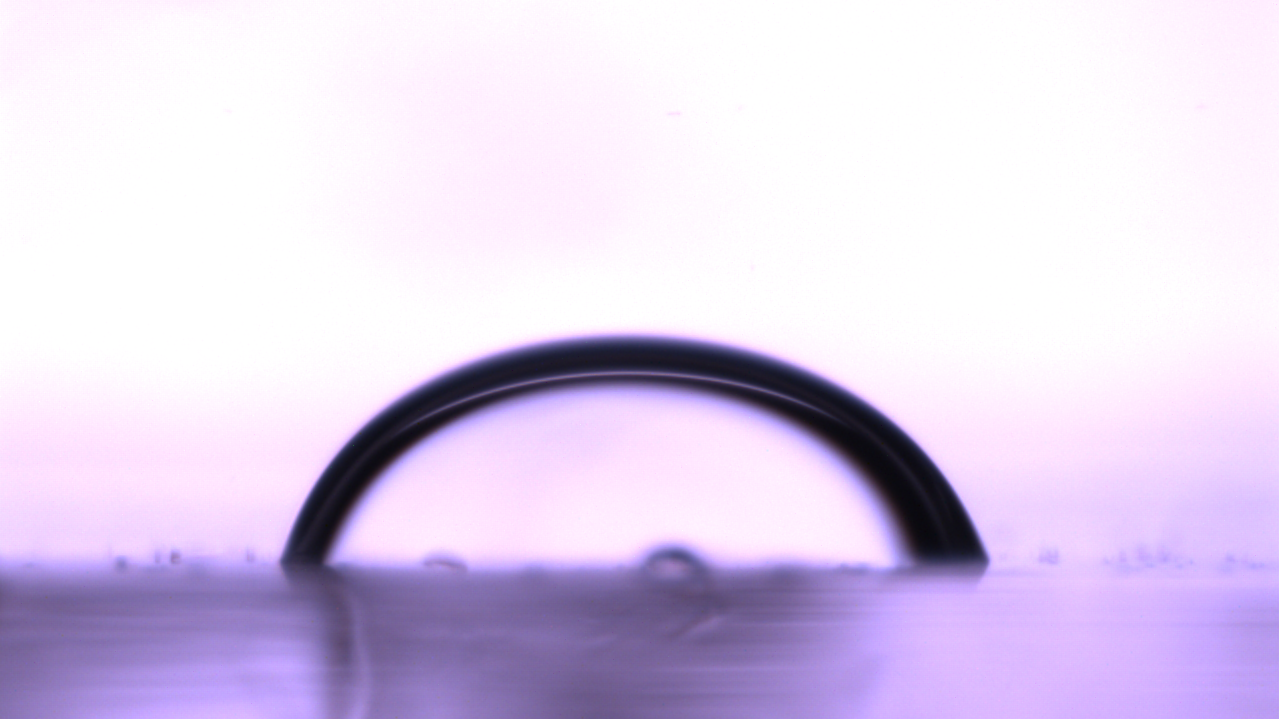

Supplement: Multimedia component 5 [file mmc5.zip › sl_pentanoic_75.22h.tif]

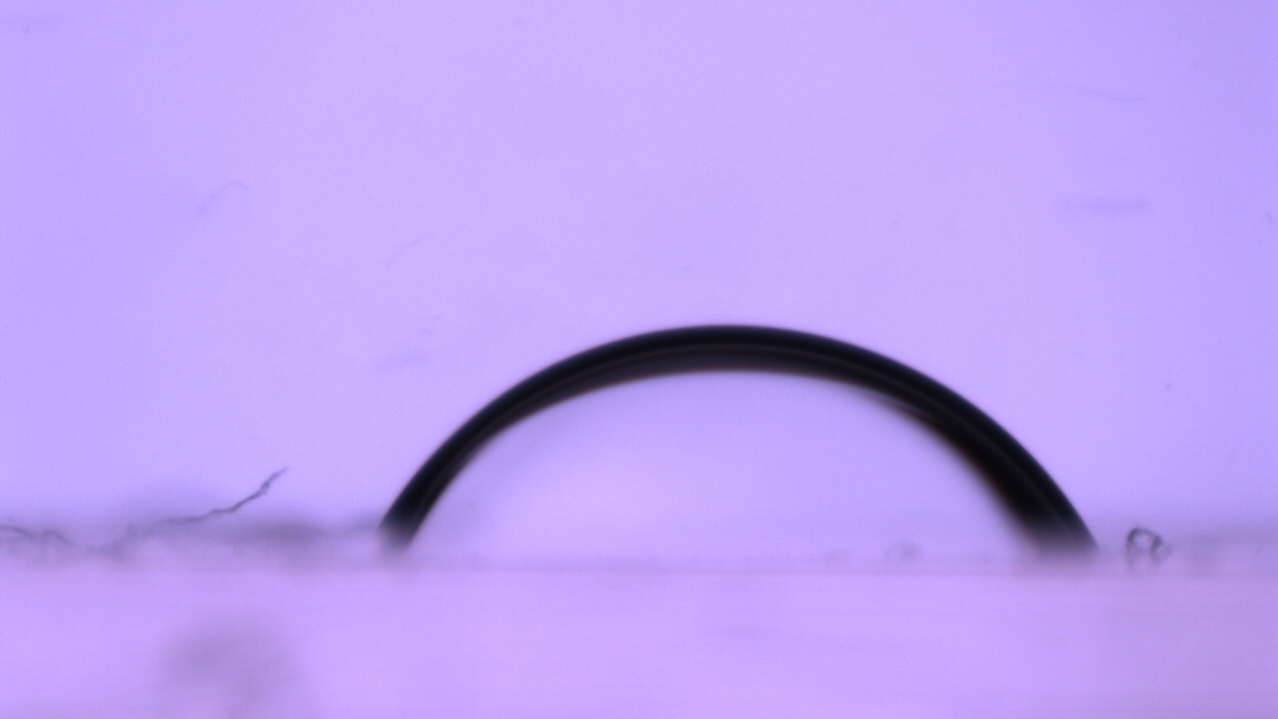

Supplement: Multimedia component 5 [file mmc5.zip › sl_pentanoic_75.35h.tif]

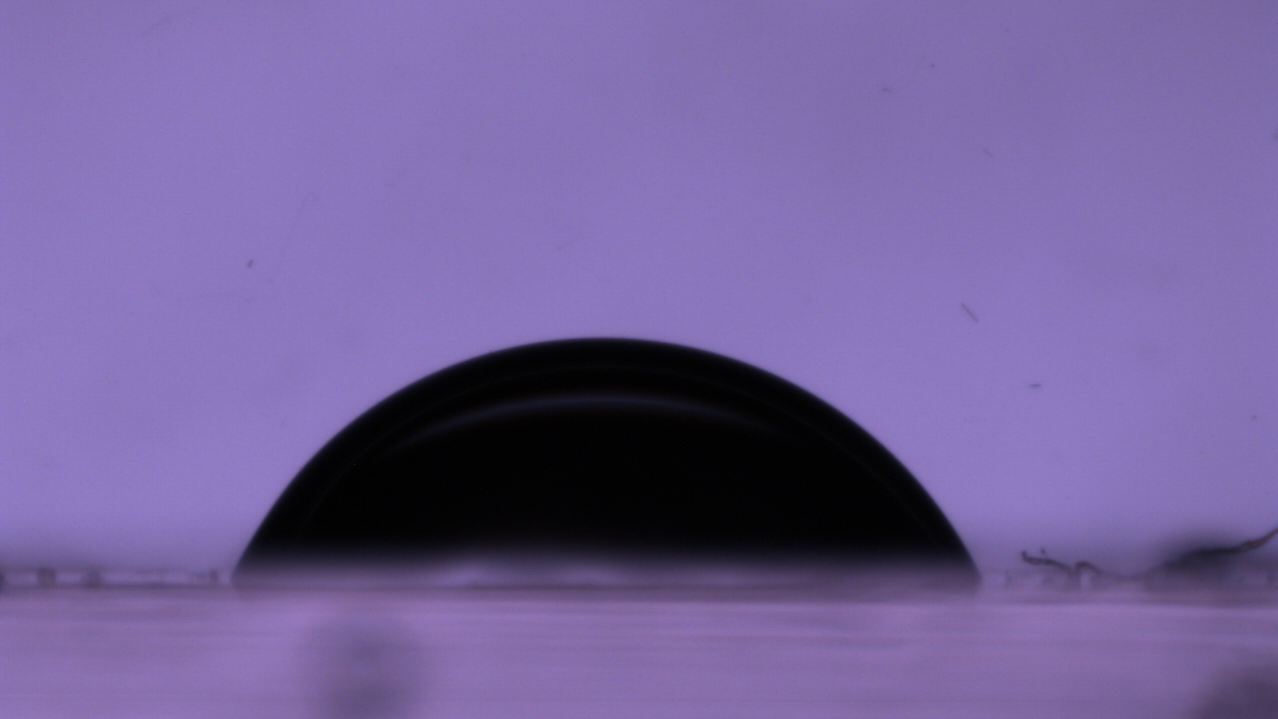

Supplement: Multimedia component 5 [file mmc5.zip › sl_pentanoic_75.40h.tif]

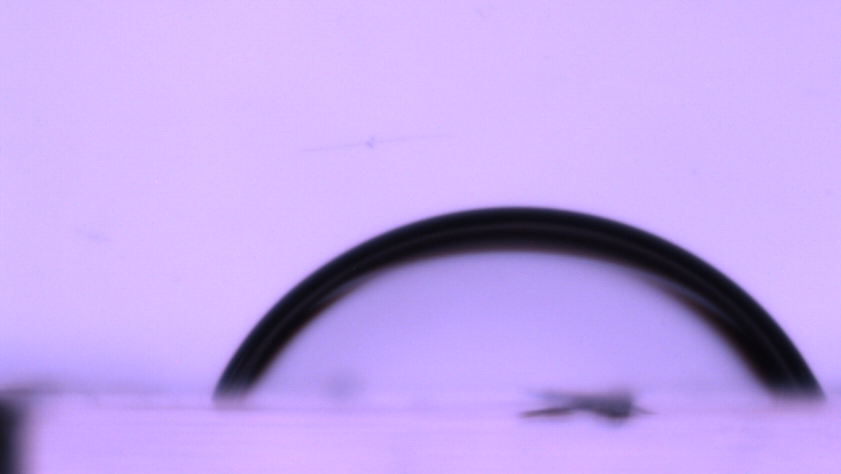

Supplement: Multimedia component 5 [file mmc5.zip › sl_pentanoic_75.43h.tif]

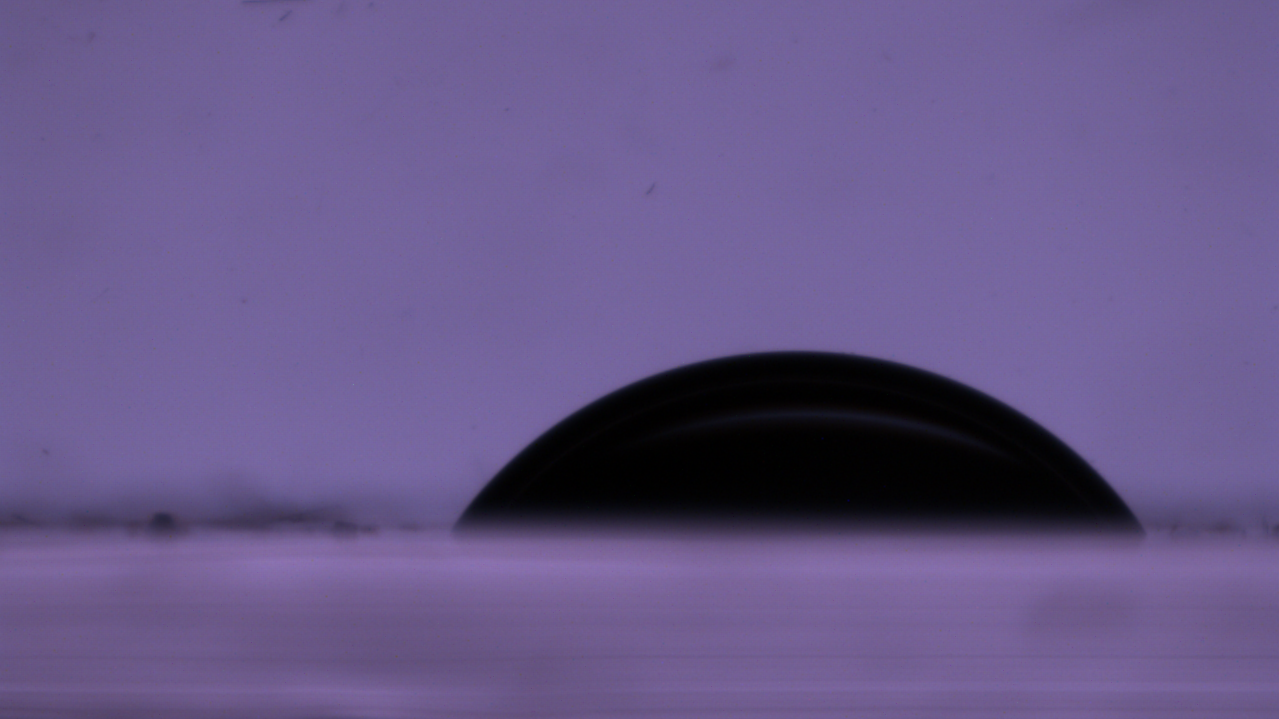

Supplement: Multimedia component 5 [file mmc5.zip › sl_pentanoic_75.50h.tif]

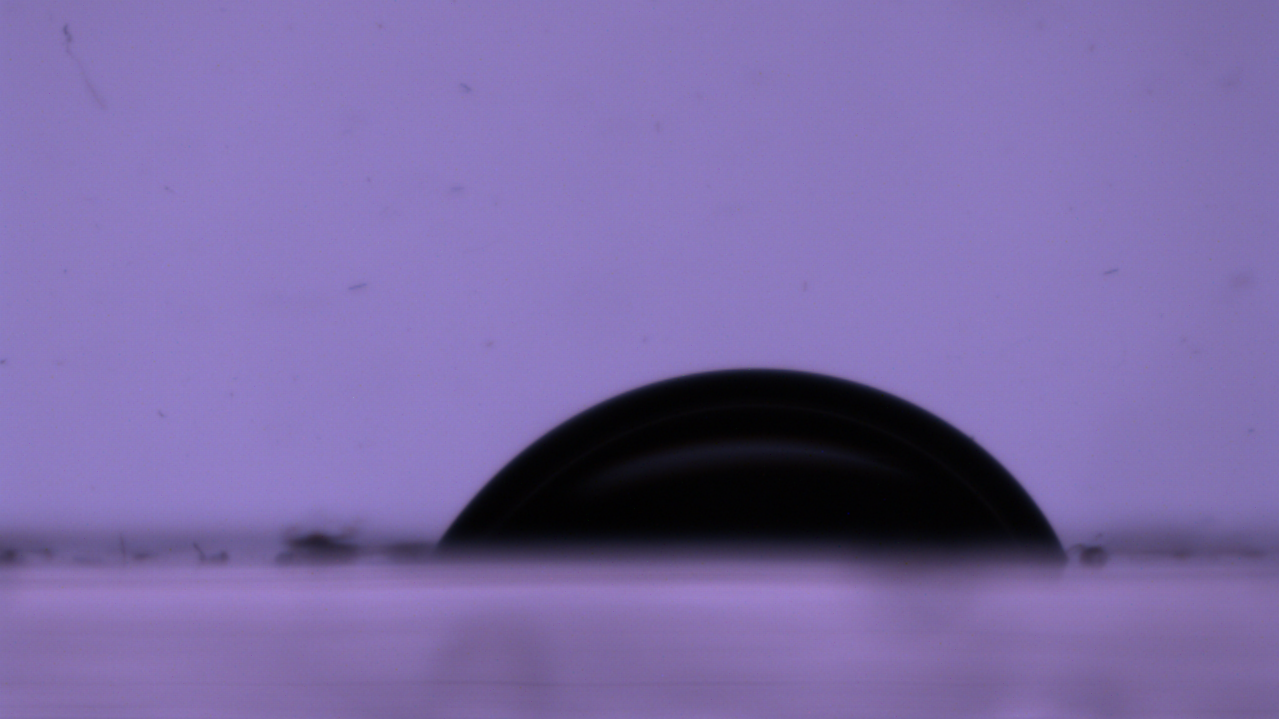

Supplement: Multimedia component 5 [file mmc5.zip › sl_pentanoic_75.53h.tif]

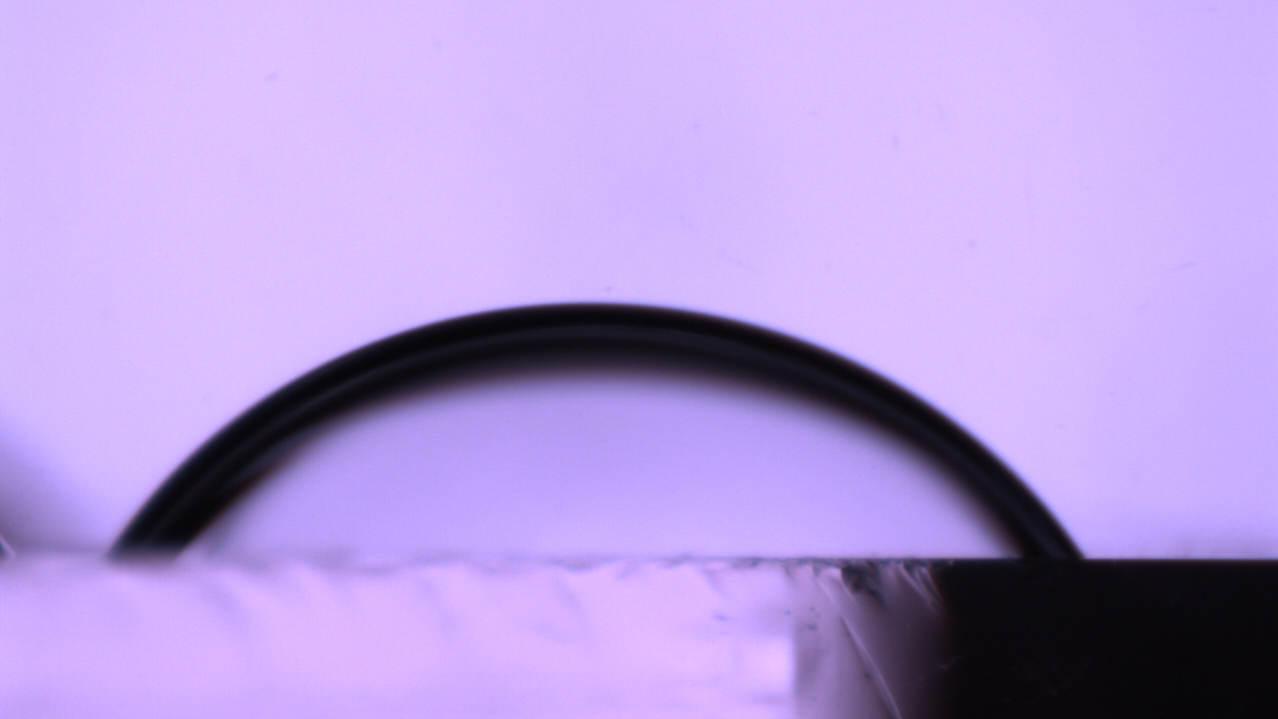

Supplement: Multimedia component 5 [file mmc5.zip › sl_pentanoic_75.62h.tif]

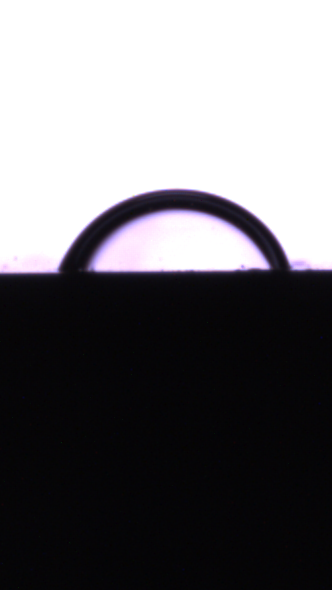

Supplement: Multimedia component 7 [file mmc7.zip › m_decane_1.02h.tif]

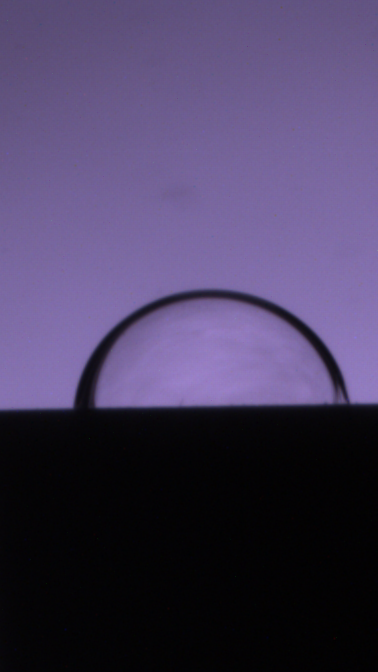

Supplement: Multimedia component 7 [file mmc7.zip › m_decane_1.10h.tif]

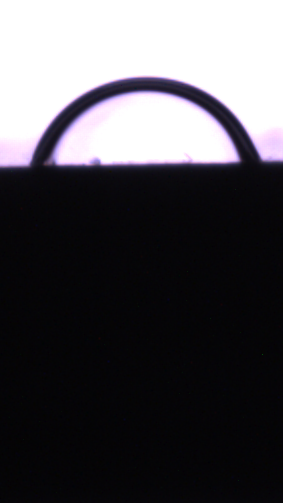

Supplement: Multimedia component 7 [file mmc7.zip › m_decane_1.30h.tif]

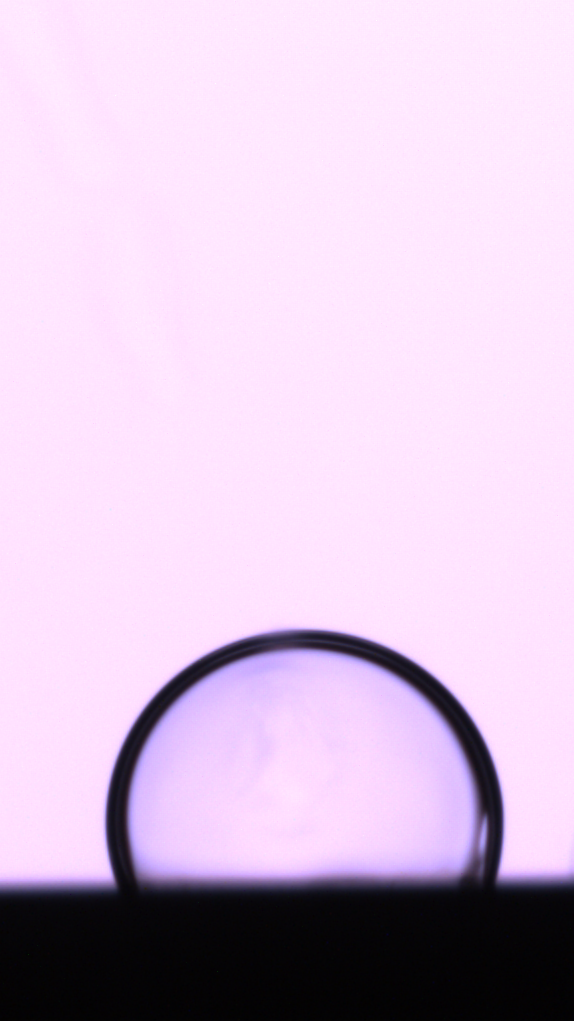

Supplement: Multimedia component 7 [file mmc7.zip › m_decane_75.00h.tif]

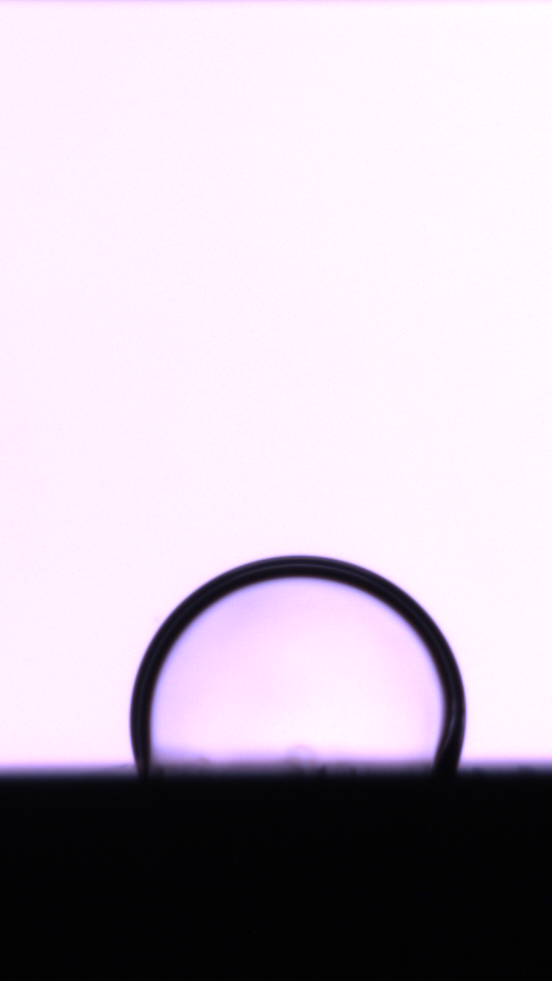

Supplement: Multimedia component 7 [file mmc7.zip › m_decane_75.35h.tif]

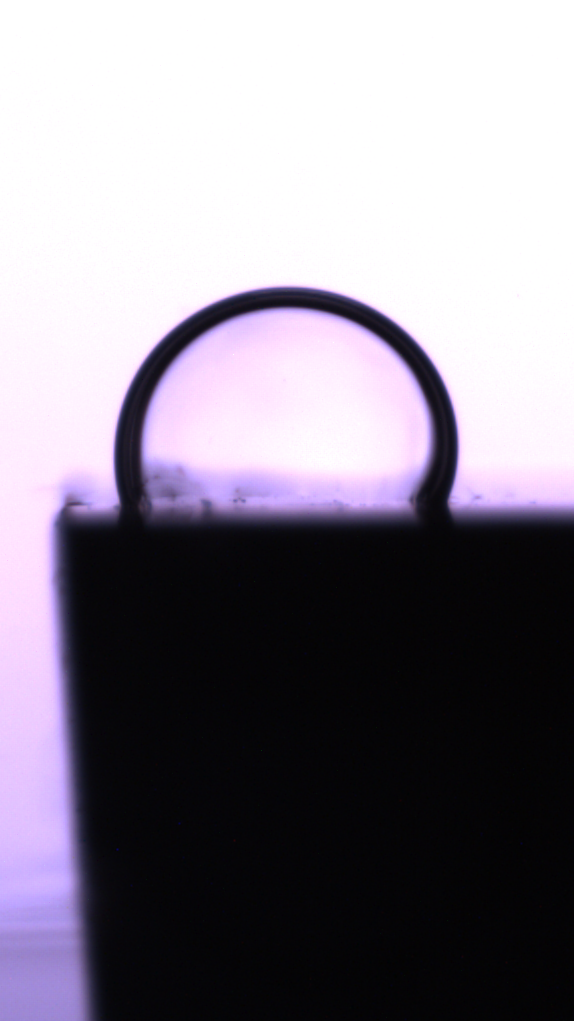

Supplement: Multimedia component 7 [file mmc7.zip › m_decane_75.50h.tif]

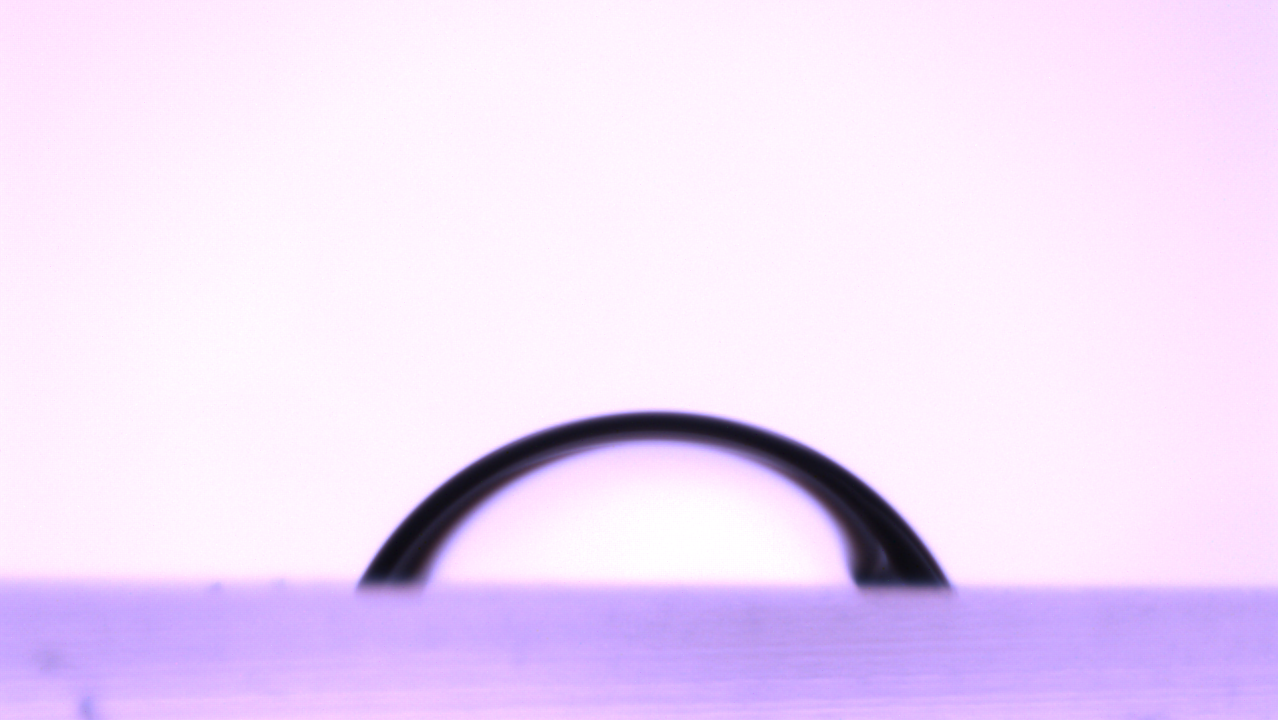

Supplement: Multimedia component 7 [file mmc7.zip › sl_decane_1.05h.tif]

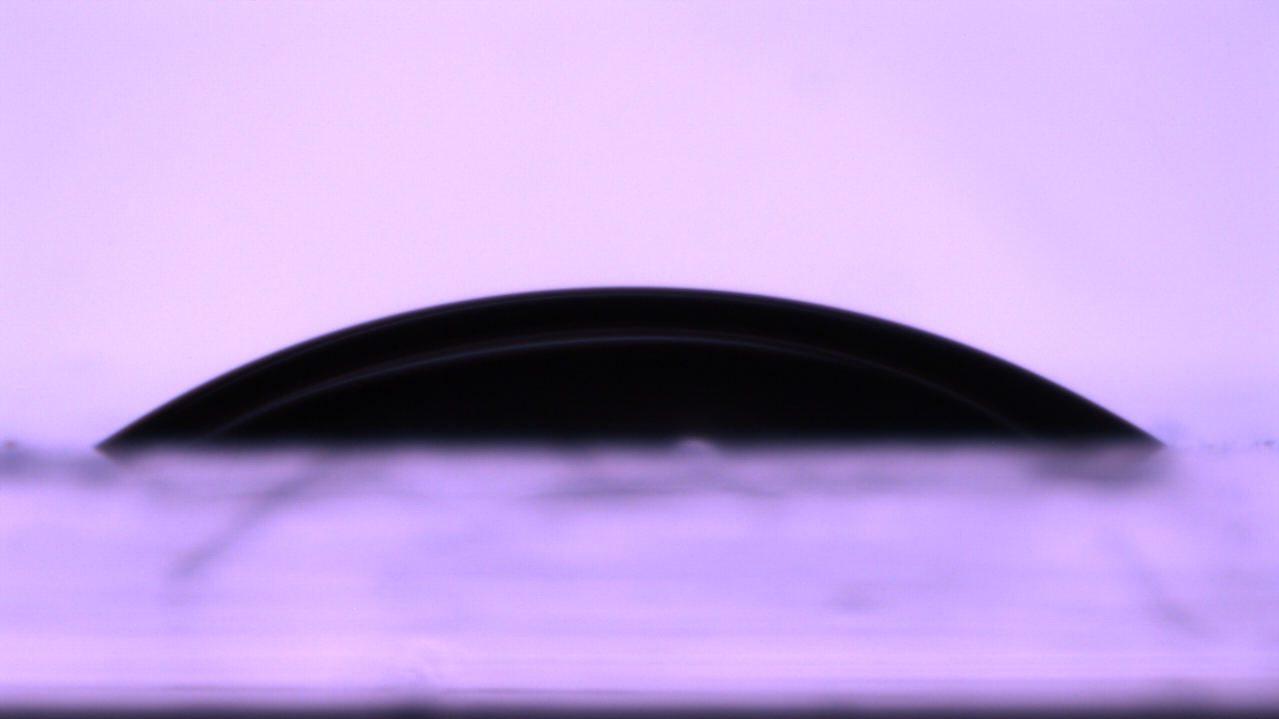

Supplement: Multimedia component 7 [file mmc7.zip › sl_decane_1.28h(2).tif]

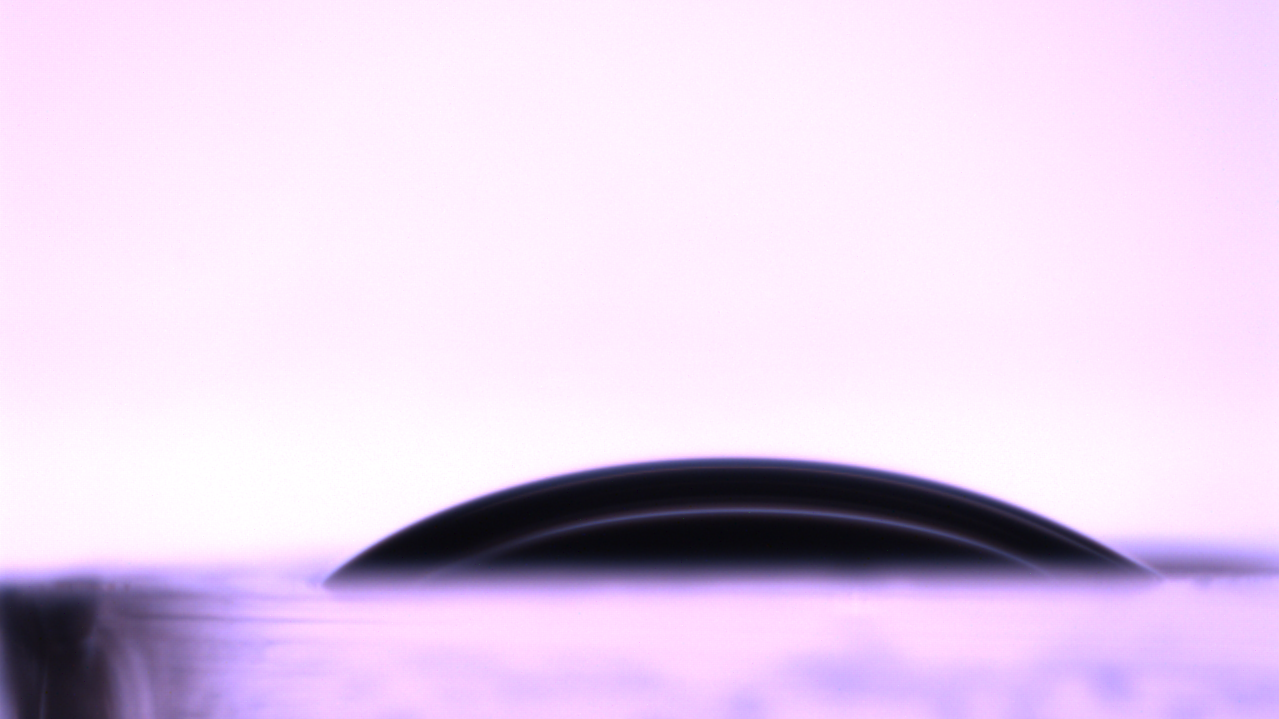

Supplement: Multimedia component 7 [file mmc7.zip › sl_decane_1.28h.tif]

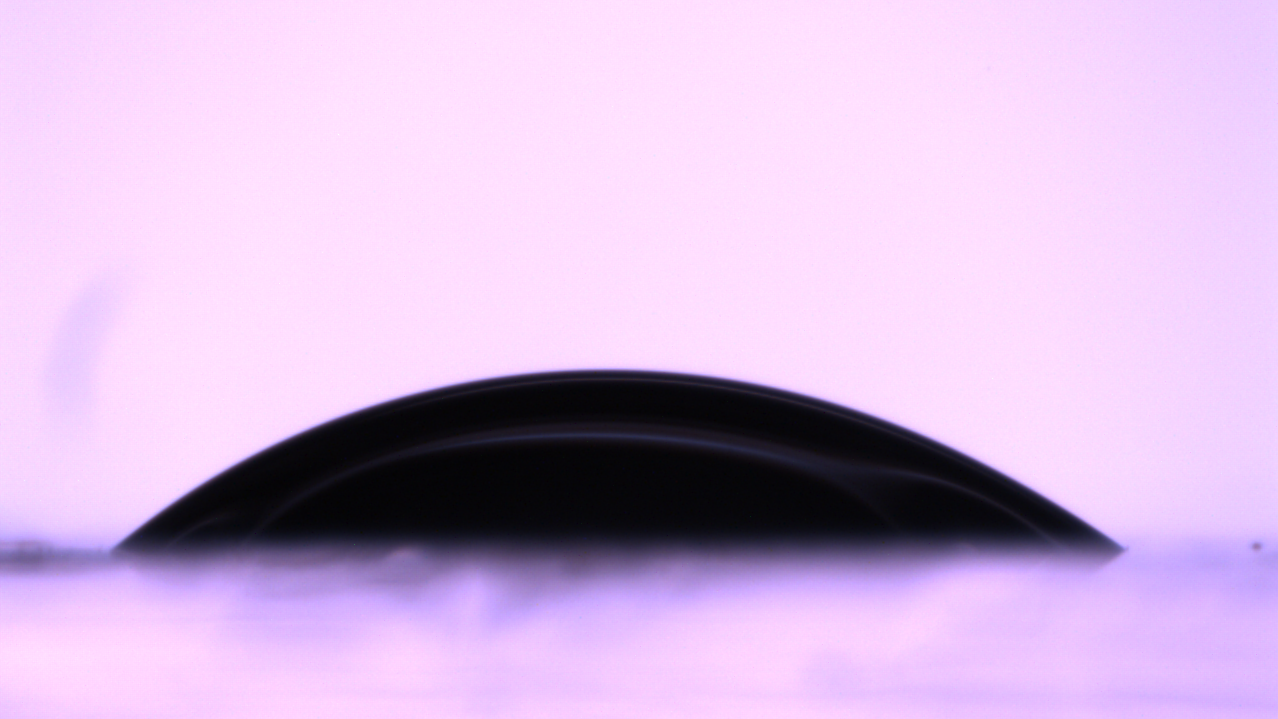

Supplement: Multimedia component 7 [file mmc7.zip › sl_decane_1.37h.tif]

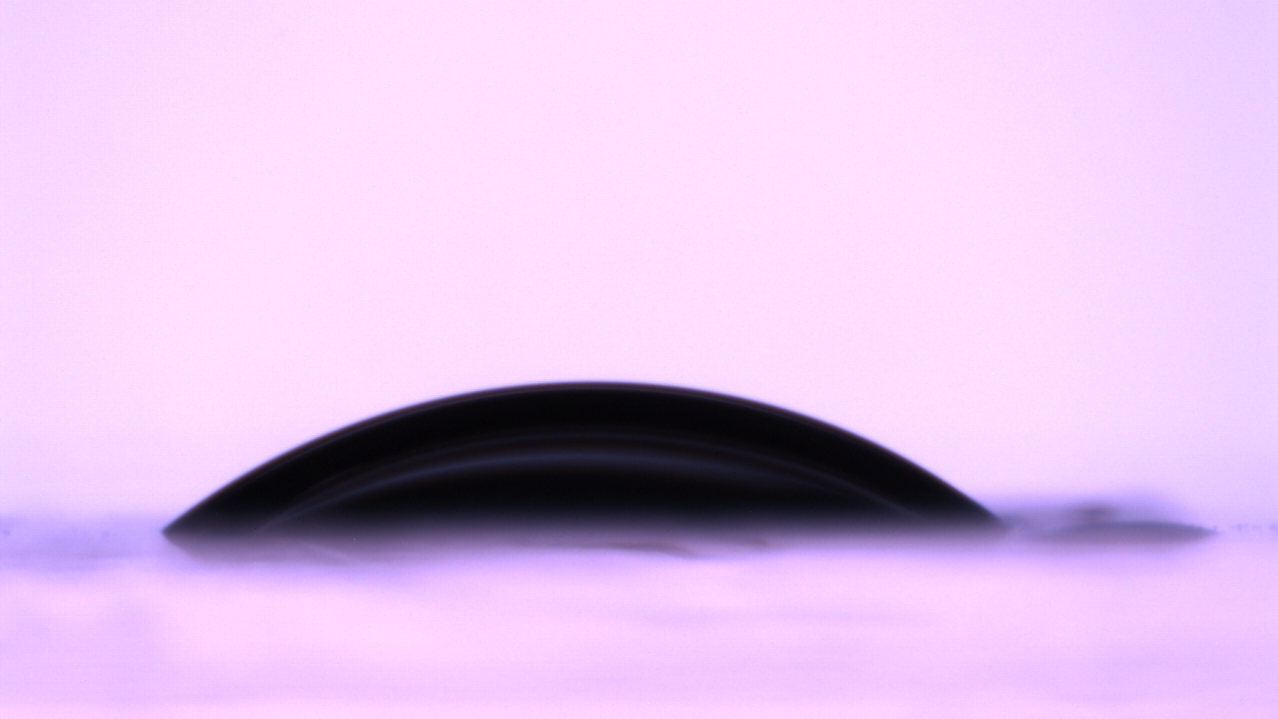

Supplement: Multimedia component 7 [file mmc7.zip › sl_decane_1.45h.tif]

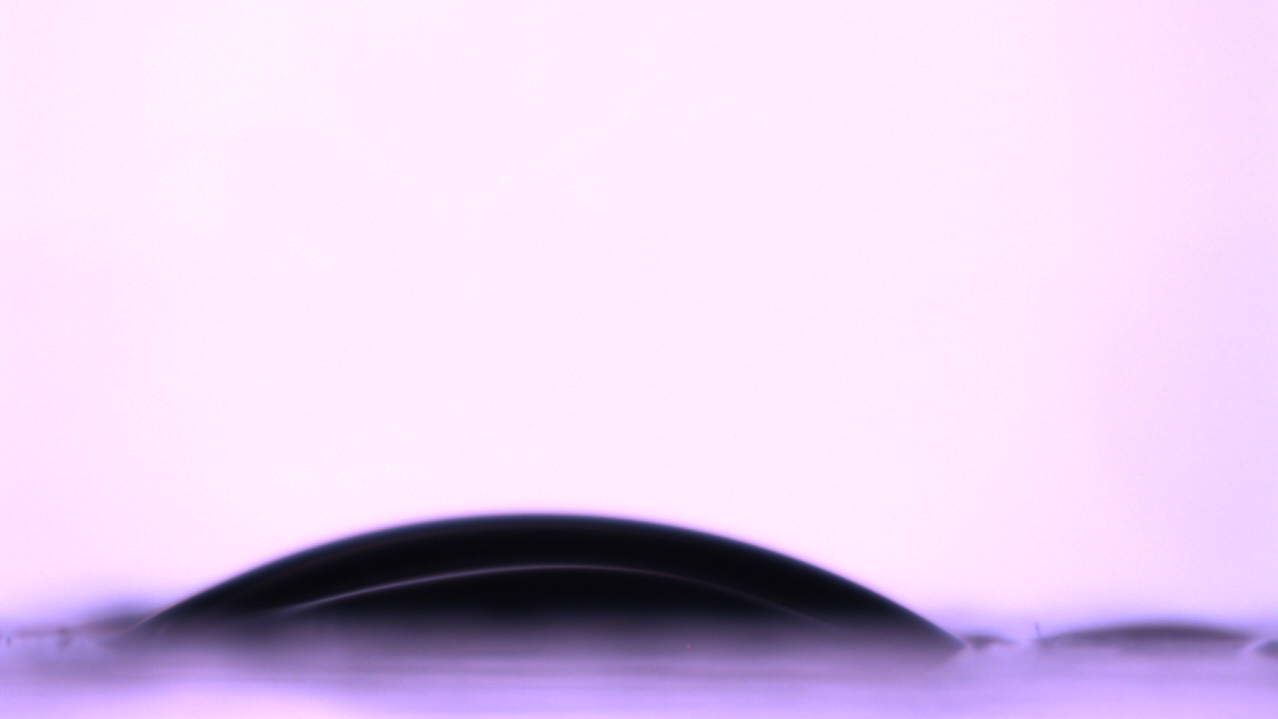

Supplement: Multimedia component 7 [file mmc7.zip › sl_decane_1.48h.tif]

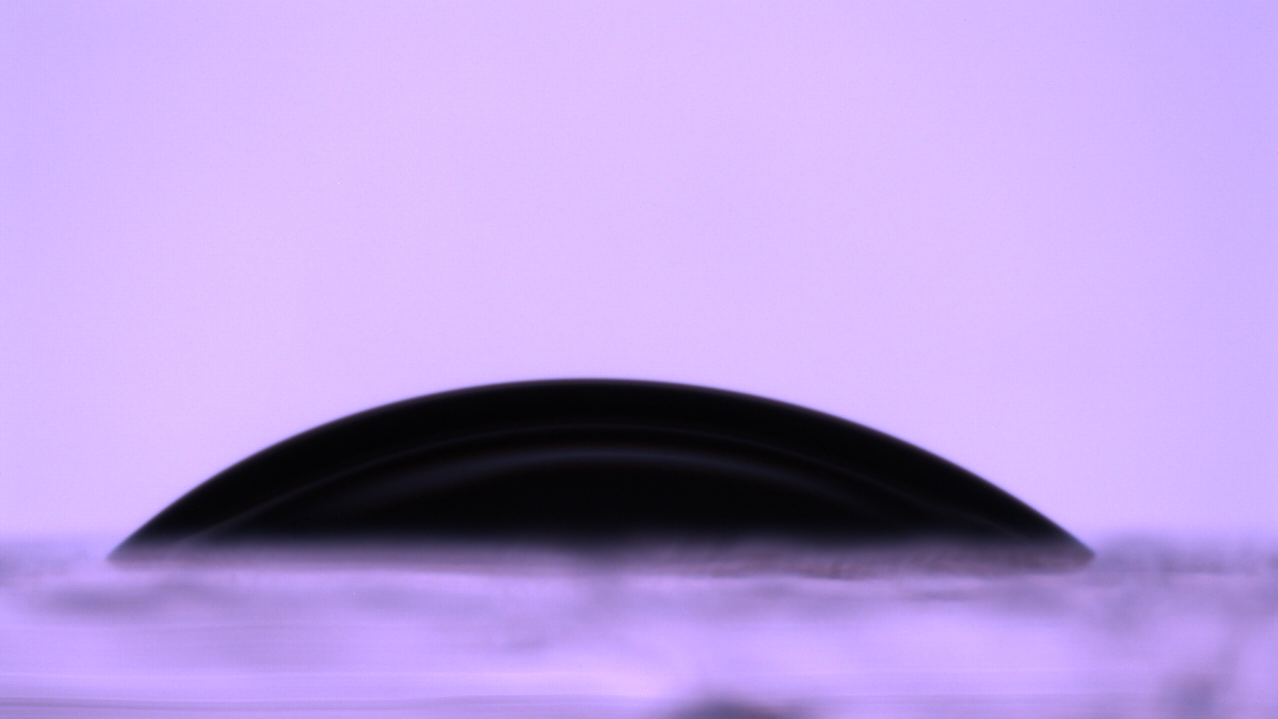

Supplement: Multimedia component 7 [file mmc7.zip › sl_decane_1.60h.tif]

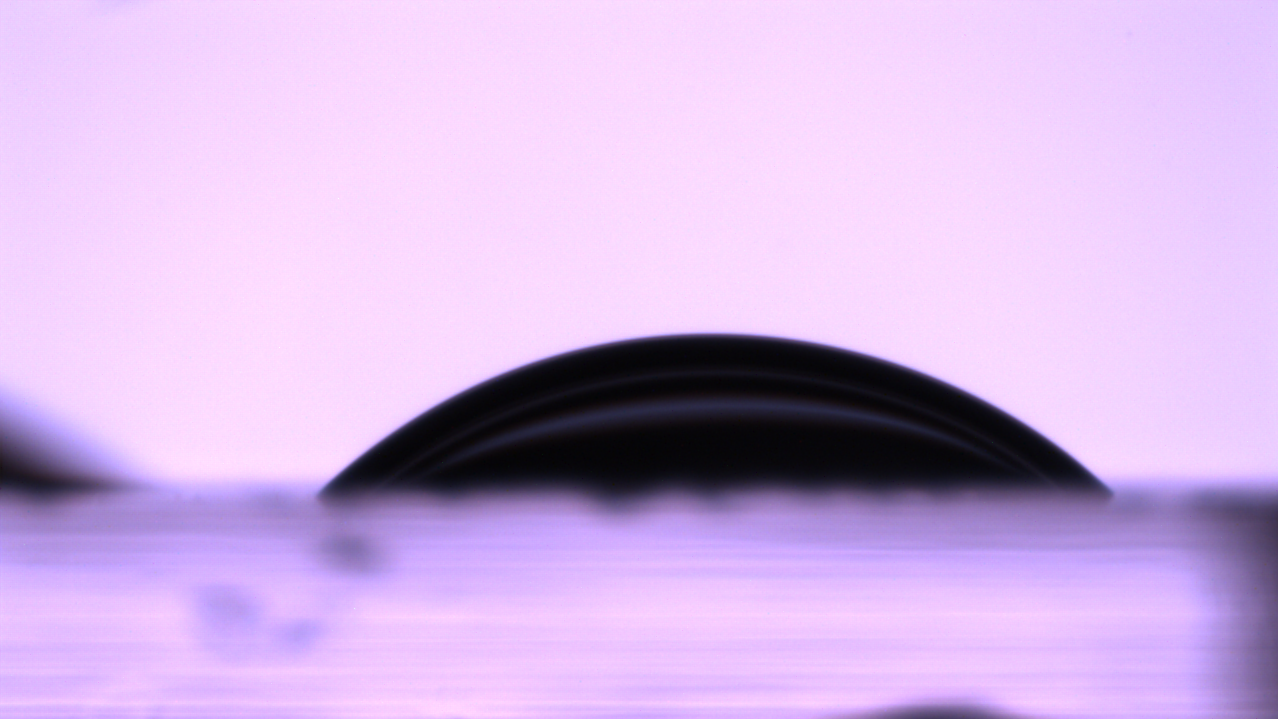

Supplement: Multimedia component 7 [file mmc7.zip › sl_decane_1.65h.tif]

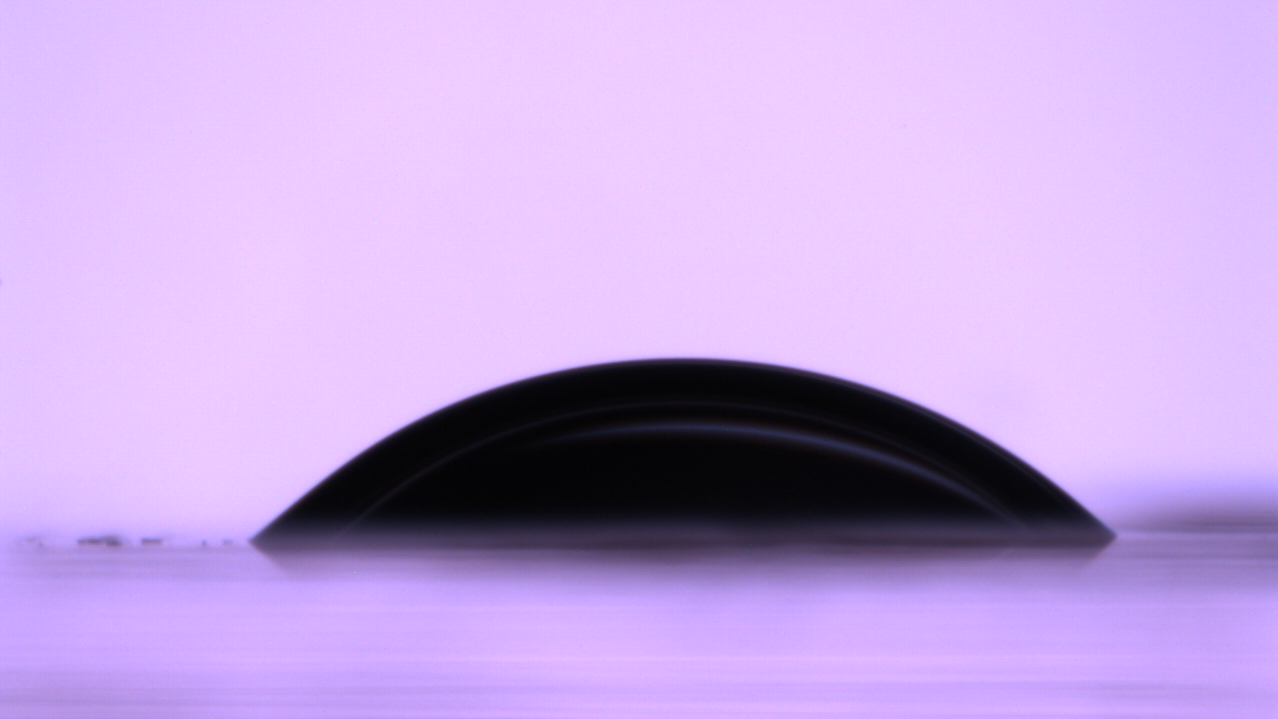

Supplement: Multimedia component 7 [file mmc7.zip › sl_decane_1.80h.tif]

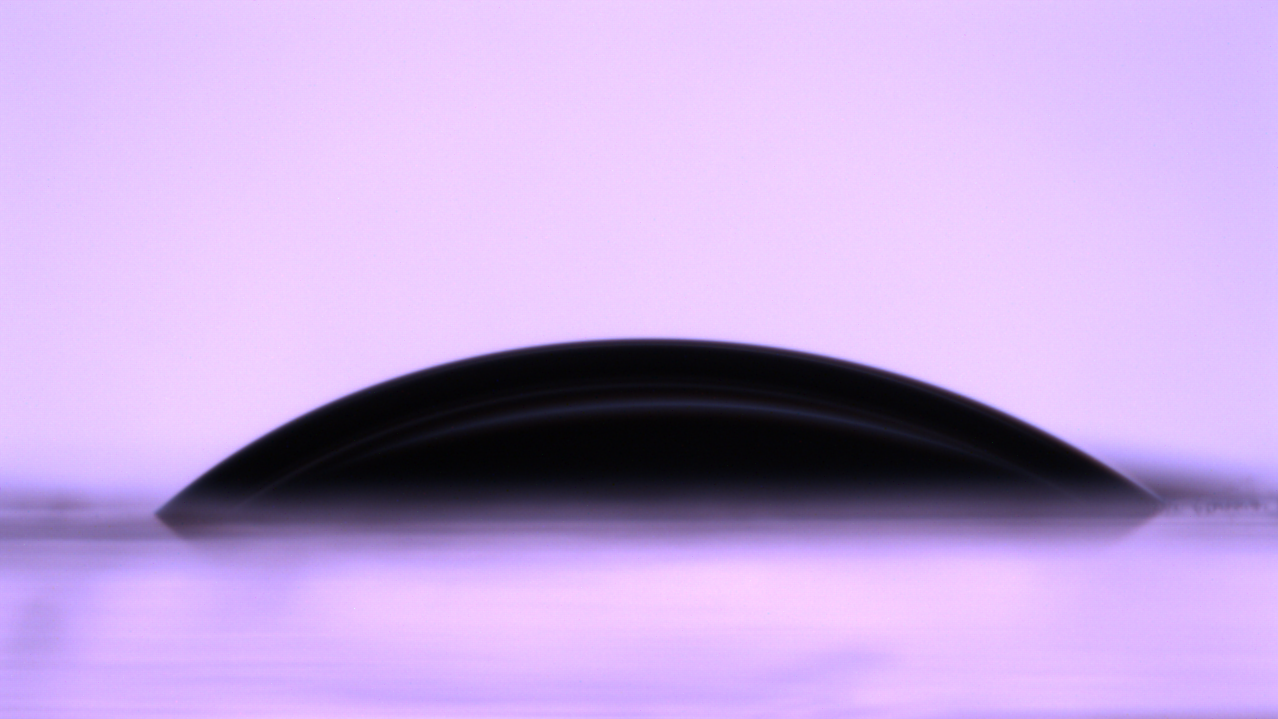

Supplement: Multimedia component 7 [file mmc7.zip › sl_decane_1.85h.tif]

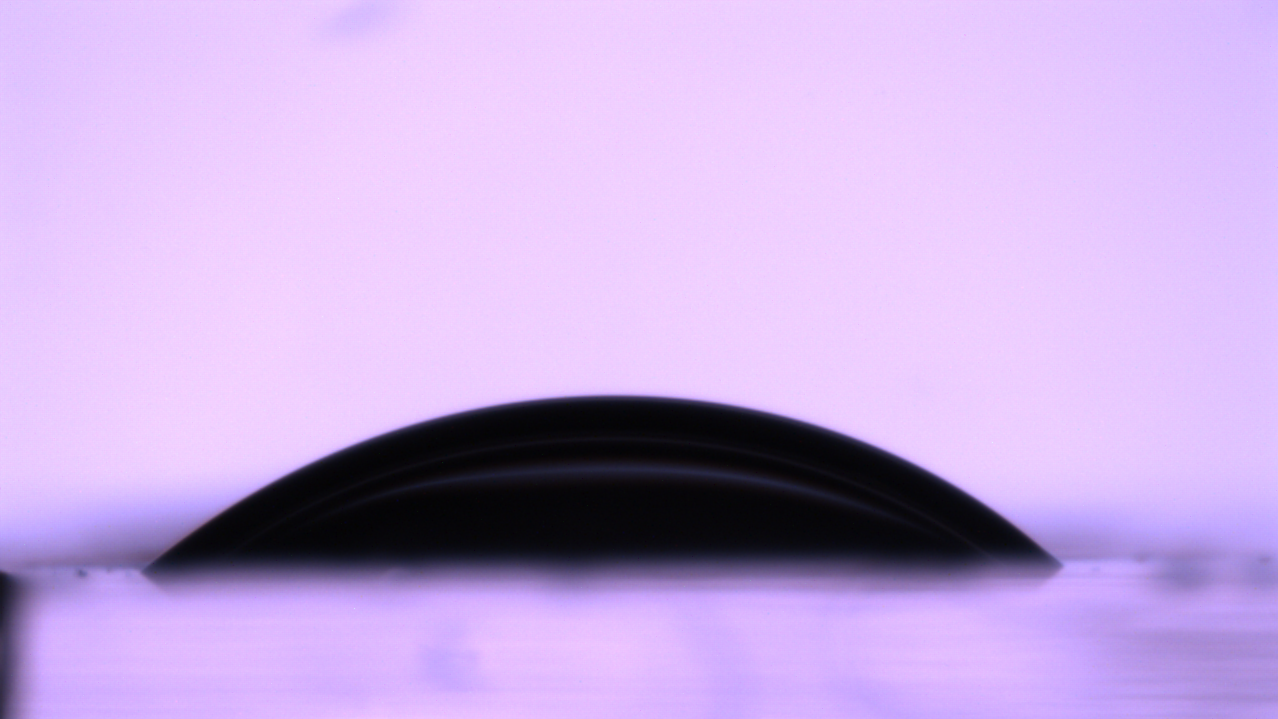

Supplement: Multimedia component 7 [file mmc7.zip › sl_decane_1.92h.tif]

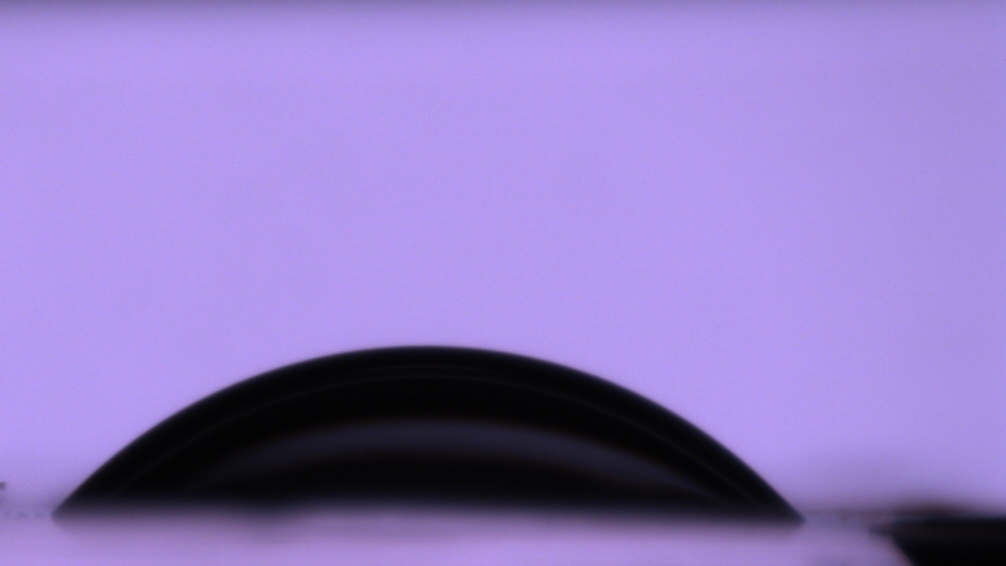

Supplement: Multimedia component 7 [file mmc7.zip › sl_decane_75.00h.tif]

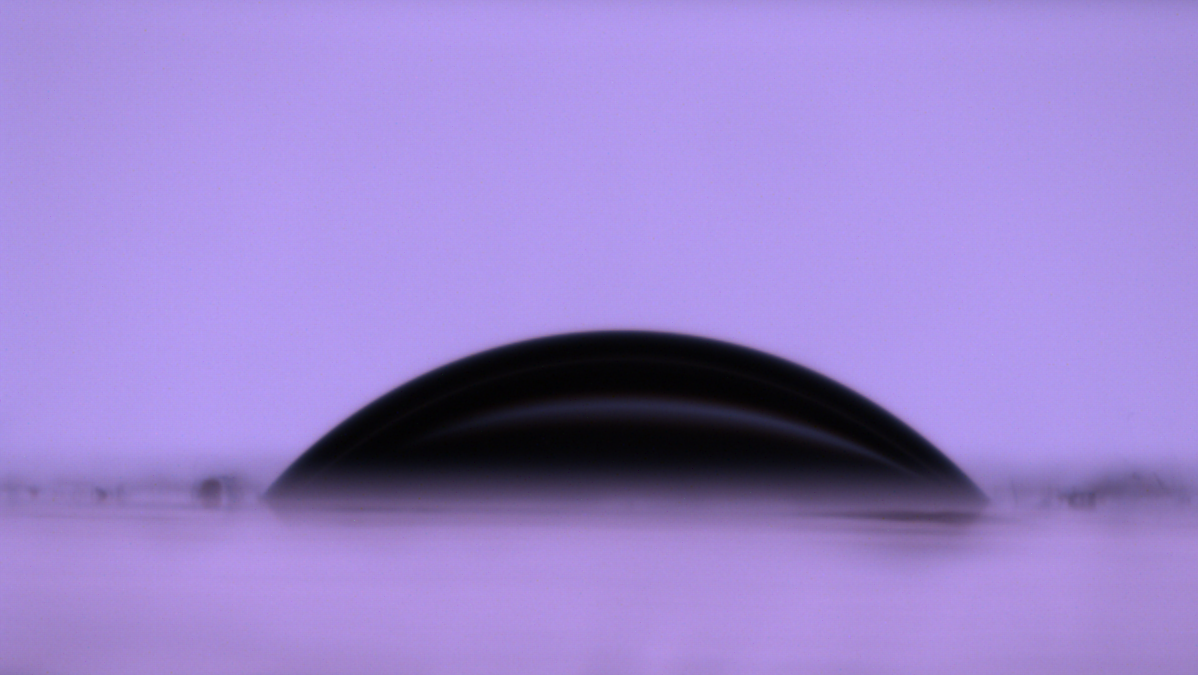

Supplement: Multimedia component 7 [file mmc7.zip › sl_decane_75.03h.tif]

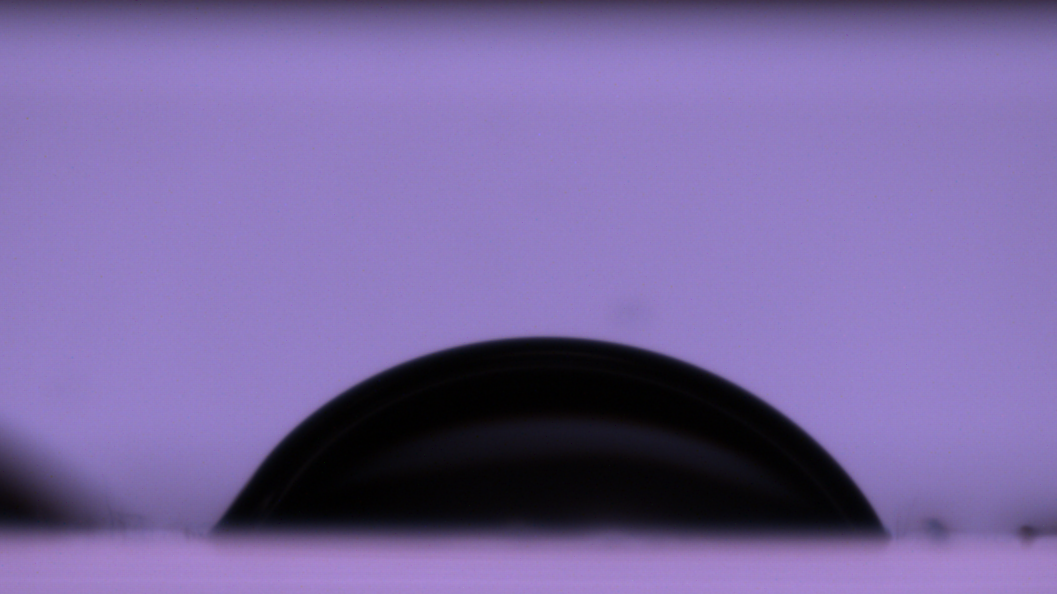

Supplement: Multimedia component 7 [file mmc7.zip › sl_decane_75.13h.tif]

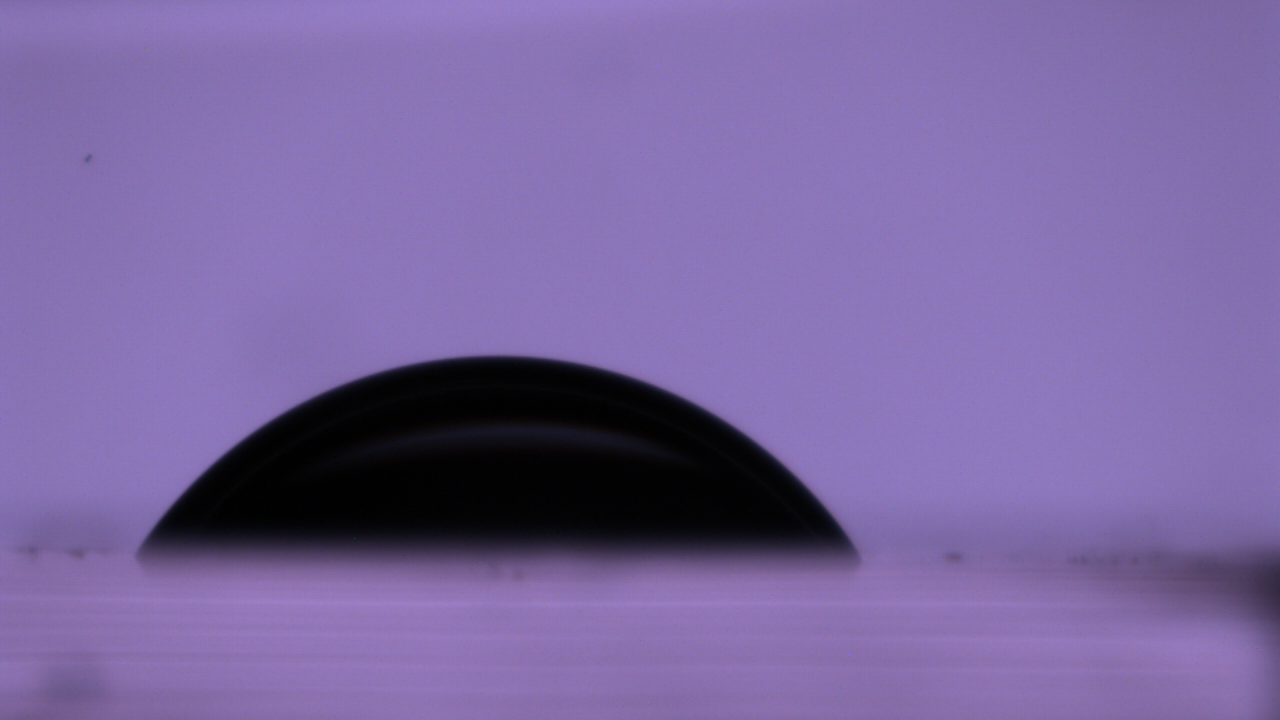

Supplement: Multimedia component 7 [file mmc7.zip › sl_decane_75.20h.tif]

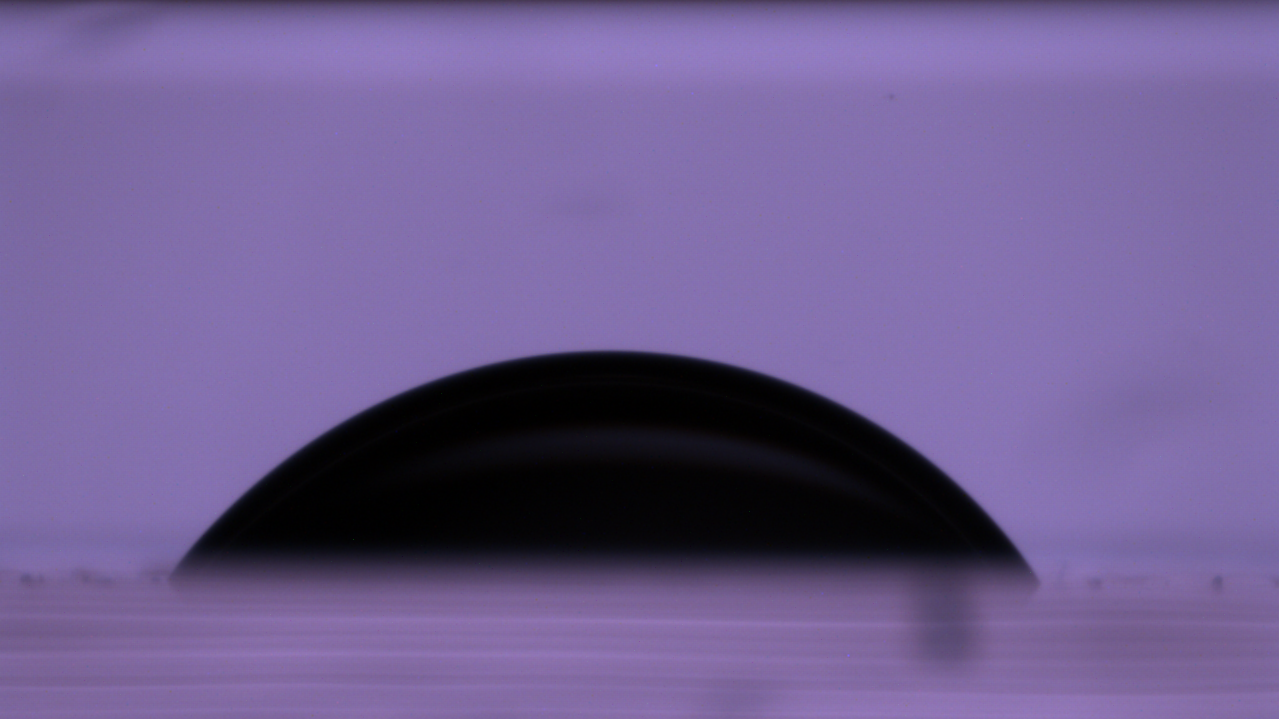

Supplement: Multimedia component 7 [file mmc7.zip › sl_decane_75.25h.tif]

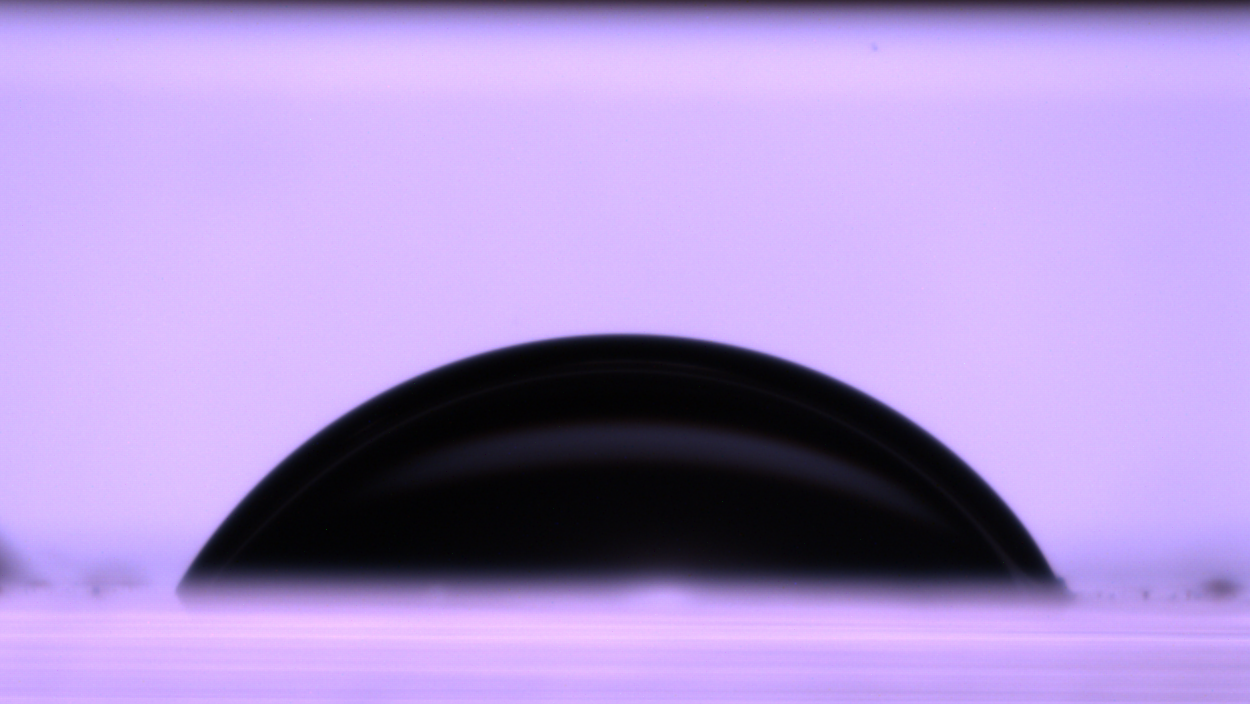

Supplement: Multimedia component 7 [file mmc7.zip › sl_decane_75.28h.tif]

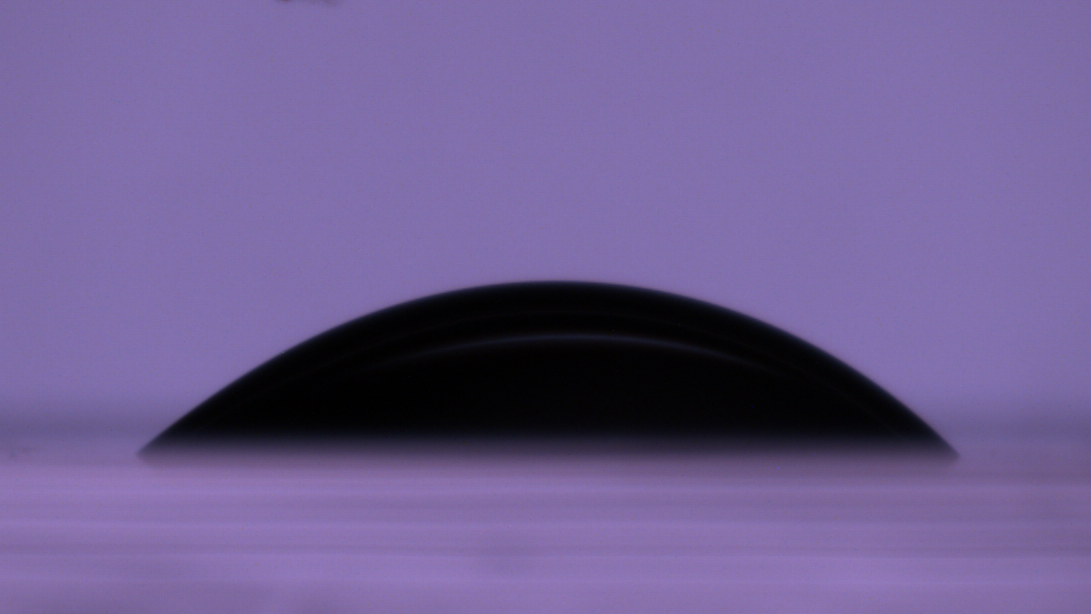

Supplement: Multimedia component 7 [file mmc7.zip › sl_decane_75.63h.tif]

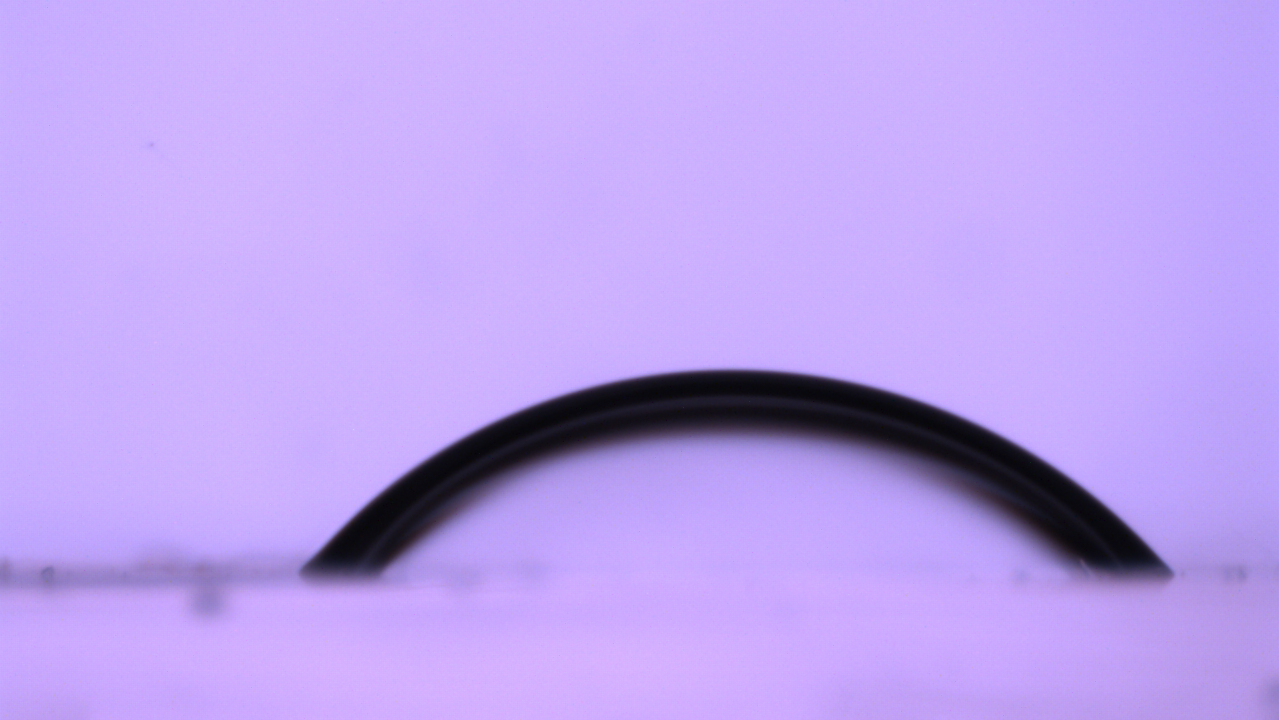

Supplement: Multimedia component 7 [file mmc7.zip › sl_decane_75.67h.tif]

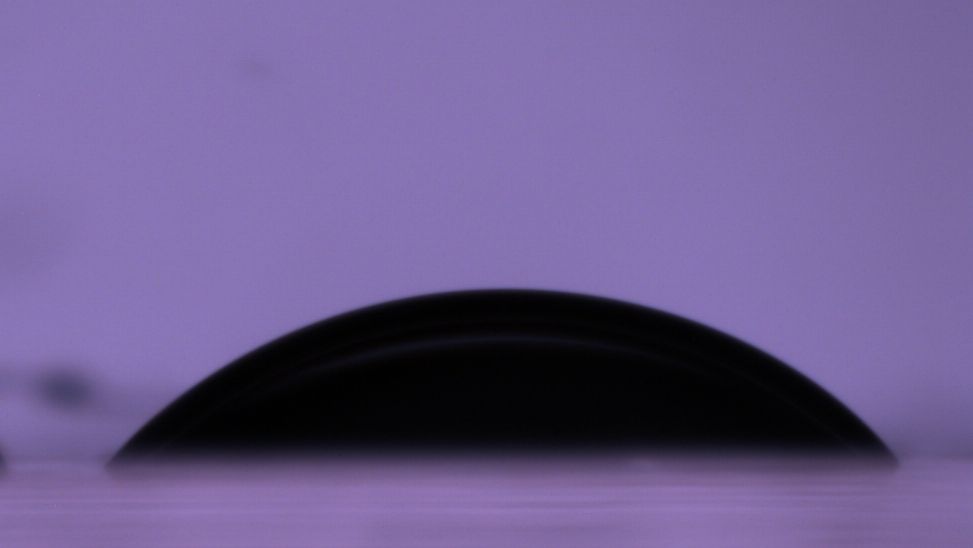

Supplement: Multimedia component 7 [file mmc7.zip › sl_decane_75.70h.tif]
